# An L,L-diaminopimelate aminotransferase mutation leads to metabolic shifts and growth inhibition in Arabidopsis

**DOI:** 10.1093/jxb/ery325

**Published:** 2018-09-12

**Authors:** João Henrique F Cavalcanti, Menny Kirma, Jessica A S Barros, Carla G S Quinhones, Ítalo A Pereira-Lima, Toshihiro Obata, Adriano Nunes-Nesi, Gad Galili, Alisdair R Fernie, Tamar Avin-Wittenberg, Wagner L Araújo

**Affiliations:** 1 Departamento de Biologia Vegetal, Universidade Federal de Viçosa, Viçosa, Minas Gerais, Brazil; 2 Max-Planck-partner group at the Departamento de Biologia Vegetal, Universidade Federal de Viçosa, Viçosa, Minas Gerais, Brazil; 3 Department of Plant Science, The Weizmann Institute of Science, Rehovot, Israel; 4 Max-Planck-Institute of Molecular Plant Physiology, Potsdam-Golm, Germany; 5 Department of Plant and Environmental Sciences, The Alexander Silberman Institute of Life Sciences, Hebrew University of Jerusalem, Givat Ram, Jerusalem Israel

**Keywords:** Alternative respiration, amino acid, carbon partition, L,L-diaminopimelate aminotransferase, lysine biosynthesis, primary metabolism

## Abstract

Lysine (Lys) connects the mitochondrial electron transport chain to amino acid catabolism and the tricarboxylic acid cycle. However, our understanding of how a deficiency in Lys biosynthesis impacts plant metabolism and growth remains limited. Here, we used a previously characterized Arabidopsis mutant (*dapat*) with reduced activity of the Lys biosynthesis enzyme L,L-diaminopimelate aminotransferase to investigate the physiological and metabolic impacts of impaired Lys biosynthesis. Despite displaying similar stomatal conductance and internal CO_2_ concentration, we observed reduced photosynthesis and growth in the *dapat* mutant. Surprisingly, whilst we did not find differences in dark respiration between genotypes, a lower storage and consumption of starch and sugars was observed in *dapat* plants. We found higher protein turnover but no differences in total amino acids during a diurnal cycle in *dapat* plants. Transcriptional and two-dimensional (isoelectric focalization/SDS-PAGE) proteome analyses revealed alterations in the abundance of several transcripts and proteins associated with photosynthesis and photorespiration coupled with a high glycine/serine ratio and increased levels of stress-responsive amino acids. Taken together, our findings demonstrate that biochemical alterations rather than stomatal limitations are responsible for the decreased photosynthesis and growth of the *dapat* mutant, which we hypothesize mimics stress conditions associated with impairments in the Lys biosynthesis pathway.

## Teaser

Impaired lysine biosynthesis in *dapat* mutant simulates a stress response culminating in metabolic reprogramming, such that alternative substrates support energy generation once carbohydrate metabolism is down-regulated.

## Introduction

**Fig. 1.**
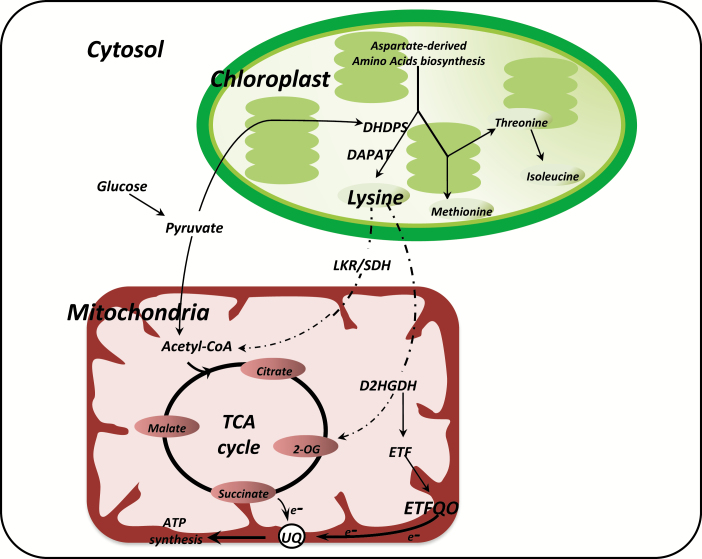
Schematic representation of lysine turnover (biosynthesis and degradation). Lysine is synthetized in the chloroplast using aspartate as a precursor. Dihydrodipicolinate synthase (DHDPS) is the first enzyme of lysine biosynthesis and it requires pyruvate export from the cytosol to the chloroplast. Under stress conditions, lysine is exported from the chloroplast to mitochondria to be degraded (trace arrows), and electrons are used as a donor for ATP synthesis in two ways: (i) lysine can be degraded by lysine-ketoglutarate reductase/saccharopine dehydrogenase (LKR/SDH) resulting in acetyl-CoA entering in the TCA cycle, or (ii) lysine can be degraded by D-2-hydroxyglutarate dehydrogenase (D2HGDH) resulting in 2-oxoglutarate (2-OG), which at the same time acts as an electron donor for the alternative respiration system mediated by electron transfer flavoprotein (ETF)–electron transfer flavoprotein:ubiquinone oxidoreductase (ETFQO). Thus there is a close relationship between chloroplasts and mitochondria in lysine metabolism.

Plant mitochondria play a pivotal role in the biosynthesis of cellular ATP through oxidative phosphorylation. The tricarboxylic acid (TCA) cycle in the mitochondria is crucial in oxidizing acetyl-CoA to produce NADH, FADH_2_, ATP, and carbon skeletons to be used in other metabolic processes (Fernie *et al.*, 2004; Millar *et al.*, 2011; Araújo *et al.*, 2012). Compelling evidence has demonstrated that plant respiration is mainly dependent on carbohydrate oxidation (Plaxton and Podesta, 2006), but under stress conditions (which affect carbohydrate supply), metabolism is altered and alternative pathways are induced to provide substrates to the respiratory processes (Ishizaki *et al.*, 2006; Araújo *et al.*, 2010; Galili *et al.*, 2014; Hildebrandt *et al.*, 2015). It has been demonstrated that protein and amino acid degradation are highly effective at sustaining leaf respiration, particularly during senescence and/or stress situations (Moller and Kristensen, 2004; Araújo *et al.*, 2011*b*; Hildebrandt *et al.*, 2015; Galili *et al.*, 2016). Additionally, protein degradation can be important for respiratory metabolism under more common physiological circumstances (Bouma *et al.*, 1994; Lehmeier *et al.*, 2008). Notably, it has been demonstrated that in Arabidopsis, lysine (Lys) degradation occurs via a branched pathway (Araújo *et al.*, 2010), partially similar to that described for the bacterium *Rhodospirillum rubrum* (Ebisuno *et al.*, 1975) and for mammalian systems (Struys and Jakobs, 2010). In this pathway, 2-hydroxyglutarate is produced via the pipecolate pathway, and branched chain keto acids are produced via an, as yet undefined, aminotransferase. It is important to note that Lys is synthesized in the chloroplasts (Fig. 1) and therefore must be transported to the mitochondria and then degraded to 2-hydroxyglutarate and further oxidized to 2-oxoglutarate (Engqvist *et al.*, 2009; Araújo *et al.*, 2010).

The electron-transfer flavoprotein (ETF)–electron-transfer flavoprotein:ubiquinone oxidoredutase (ETFQO) complex has been shown to be highly induced at a transcriptional level during dark-induced senescence (Buchanan-Wollaston *et al.*, 2005) and oxidative stress (Lehmann *et al.*, 2009), as well as under conditions in which free amino acids are present at high concentrations (Weigelt *et al.*, 2008). By analysing select Arabidopsis mutants using enzymatic, metabolic, and isotope-labelling procedures it was demonstrated that the substrates used by the ETF–ETFQO pathway [mainly the branched-chain amino acids (BCAAs) isoleucine, leucine, and valine, as well as Lys] represent alternative electron donors at the mitochondrial level (Engqvist *et al.*, 2009; Araújo *et al.*, 2010). This donation occurs either directly, by the transfer of electrons to the mitochondrial electron transport chain (mETC) via the ETF complex, or indirectly, by the supply of substrates to fuel the TCA cycle (Araújo *et al.*, 2010). To date, only two alternative dehydrogenases [isovaleryl-CoA dehydrogenase (IVDH) and D-2-hydroxyglutarate dehydrogenase (D2HGDH)] have been demonstrated to be able to donate electrons to the ETF–ETFQO system in plants (Araújo *et al.*, 2010). Accordingly, Lys catabolism via either D2HGDH or IVDH suggests a potential connection between the TCA cycle and alternative respiration in the maintenance of energy metabolism. This fact supports the growing evidence of strong network behaviour in the co-ordination of amino acid metabolism (Sweetlove and Fernie, 2005; Less and Galili, 2008; Araújo *et al.*, 2010; Gu *et al.*, 2010). Taken together, this information reinforces the idea that Lys metabolism has a strong correlation not only with the TCA cycle but also with mitochondrial energy metabolism in general (Angelovici *et al.*, 2011; Kirma *et al.*, 2012; Galili and Amir, 2013).

Compelling evidence has demonstrated that composite branched pathways are generally responsible for the biosynthesis of amino acids, and in particular the branched aspartate metabolic network has been extensively studied (Karchi *et al.*, 1994; Zhu and Galili, 2003; Angelovici *et al.*, 2009; Less and Galili, 2009; Angelovici *et al.*, 2011; Clark and Lu, 2015). By contrast, little is currently known concerning the biological impact of a deficiency in the biosynthesis of aspartate-family amino acids, as naturally occurs in response to stress (Less and Galili, 2008; Baena-González and Sheen, 2008). Interestingly, an Arabidopsis mutant selected for enhanced defence against *Pseudomonas syringae* (Rate and Greenberg, 2001; Song *et al.*, 2004) was further demonstrated to be unequivocally caused by a single amino acid substitution in the L,L-diaminopimelate aminotransferase (LL-DAPAT) enzyme of Lys biosynthesis, significantly reducing its activity (Hudson *et al.*, 2006). Coupling bioinformatics with elegant biochemical results, it has been demonstrated that plants use a variant of the bacterial pathway for Lys production mediated uniquely by LL-DAPAT (Hudson *et al.*, 2005). Remarkably, this discovery in Arabidopsis demonstrated the presence of an unnoticed mechanism for Lys synthesis in nature (McCoy *et al.*, 2006). The mutation in the LL-DAPAT gene (AT4G33680) also resulted in dwarfism, altered leaf morphology and enhanced accumulation of the stress hormone salicylic acid (SA), and was therefore originally named the ‘*Aberrant Growth and Death*’ (*agd2*) mutant (Rate and Greenberg, 2001; Song *et al.*, 2004).

Here, we investigated the metabolic and physiological impact of impaired Lys biosynthesis by using this established Arabidopsis mutant (hereafter referred to as *dapat*) with reduced activity of the Lys biosynthesis enzyme LL-DAPAT. Our results demonstrate that the mutation in the LL-DAPAT gene culminated in growth impairments coupled with decreases in photosynthesis and maintenance of respiration. Furthermore, transcriptomic, proteomic, and metabolic analyses are suggestive of an imbalance in diel carbon and nitrogen metabolism. We additionally investigated the metabolic response of other mutants involved in amino acid metabolism, transport, or signalling to demonstrate that metabolite changes are triggered specifically by the DAPAT mutation.

## Materials and methods

### Plant material and growth conditions

Arabidopsis wild-type (WT) and the *dapat* [previously referred to as *aberrant growth death 2* (*agd-2*) characterized by Rate and Greenberg (2001)] plants used in this study were both of the Col ecotype (Col-0) (for further details see Rate and Greenberg, 2001). Seeds were surface-sterilized and imbibed for 2 d at 4 °C in the dark on 0.8% (w/v) agar plates containing half-strength Murashige and Skoog medium (Sigma-Aldrich; pH 5.7). Seeds were subsequently germinated and grown at 22 °C under short-day conditions (10 h light/14 h dark) with 150 µmol photons m^−2^ s^−1^. For phenotype examination, agar-initiated seedlings were subsequently transferred to soil 7–10 d after germination and placed in a growth chamber under similar growth conditions as above. The whole rosette leaves of 4-week-old plants were harvested for subsequent analysis.

We additionally used the following mutant lines: (i) *dhdps-2* (At2g45440), a T-DNA insertional line in the gene encoding dihydrodipicolinate synthase in the Wassilewskija (WS) background, which displays relatively lower Lys synthesis but consequently a strongly enhanced threonine synthesis (Craciun *et al.*, 2000); (ii) *kin10* (At3g01090), a line carrying a mutation in the evolutionarily conserved protein kinase that targets a remarkably broad array of genes that orchestrate transcription networks, promoting catabolism and suppressing anabolism (Baena-González *et al.*, 2007), and (iii) *lht1-1* (At5g40780), a Lys- and His-specific amino acid transporter (Hirner *et al.*, 2006). All genotypes used here were cultivated under the same conditions described above and harvested at the same time of day in order to allow proper comparison.

### L,L-Diaminopimelate aminotransferase enzyme activity assay

DAPAT activity was analysed with the *O*-aminobenzaldehyde (OAB) assay described in Hudson *et al* (2006). Frozen leaf material was ground into fine powder using a ball mill. Proteins were extracted in 100 mM HEPES–KOH (pH 7.6) from 20 mg of leaf material by vortexing. Following centrifugation at 22000 *g* for 15 min at 4 °C, the supernatant was applied to an Amicon Ultra 30000 MWCO filter unit (Millipore) for buffer exchange and concentration. The crude extract was concentrated by centrifugation at 14000 *g* for 30 min at 4 °C and diluted with 450 µl of fresh 100 mM HEPES–KOH (pH 7.6) twice. Protein concentration in the concentrated crude extract was determined by Bradford protein assay kit (Bio-Rad). DAPAT activity was measured as the increase of absorbance at 440 nm in 1 ml of 100 mM HEPES–KOH (pH 7.6), 0.5 mM L,L-diaminopimelate (Sigma-Aldrich, 89469), 2 mM 2-oxoglutarate and 1.25 mg ml^−1^ OAB (Sigma-Aldrich, A9628) following addition of concentrated crude extract.

### Measurement of photosynthetic parameters

Leaf gas exchange measurements were performed with an open-flow gas exchange system (LI-6400 XT Li-Cor Inc., Lincoln, NE, USA). The net carbon assimilation rate (*A*), stomatal conductance to water vapour (*g*_s_), and internal-to-ambient CO_2_ concentration ratio (*C*_i_/*C*_a_) were determined after at least 2 h illumination. The reference CO_2_ concentration was set at 400 μmol CO_2_ mol^−1^ air and gas exchange was determined under 150 µmol photons m^–2^ s^–1^ at the leaf level of photosynthetically active photon flux density (PPFD). All measurements were performed at 25 °C and vapour pressure deficit was maintained at 2.0 ± 0.2 kPa, whilst the amount of blue light was set to 10% PPFD to optimize stomatal aperture. For dark respiration measurements, plants were adapted for at least 30 min in the dark to avoid light-enhanced dark respiration.

### Biochemical assays

Sampling was performed in the last hour of the day (end of the day; ED), or night (end of the night; EN). For all analyses, whole rosette leaves were collected, flash-frozen in liquid nitrogen and stored at −80 °C until analysed. Each replicate represented the mean of three determinations on the same sample. Chlorophyll, total protein, total free amino acid, and nitrate contents were determined as previously described by Sienkiewicz-Porzucek *et al.* (2010). Malate and fumarate contents were determined as described by Nunes-Nesi *et al.* (2007) and the levels of starch, sucrose, glucose, and fructose were determined as described by Fernie *et al.* (2001).

### RNA extraction and microarray analysis

100 mg of harvested rosette leaves were used for total RNA extraction as described previously (Ruuska and Ohlrogge, 2001). Total RNA was treated with DNAase RQ-1 (Promega) and then RNA was amplified using two-cycle Affymetrix labelling, using the standard Affymetrix protocol. Hybridization, labelling, scanning, and data extraction were performed following standard Affymetrix protocols. Transcriptome analysis and annotation was performed using Partek© software (www.partek.com). Pre-processing was carried out using the Robust Microarray Averaging algorithm (Irizarry *et al.*, 2003). Two-way ANOVA was performed and step-up correction was applied to correct from multiple comparisons (Hochberg and Benjamini 1990). Differentially expressed genes were chosen according to step-up correction value <0.05 and a fold change >1.5 between genotypes. Over-representation analysis (enrichment) of the differently expressed genes was performed on PageMan (http://mapman.mpimp-golm.mpg.de/general/ora/ora.shtml) (Usadel *et al.*, 2006), using Fisher’s exact test suggested in PageMan for enriched categories. Visualization of metabolic pathways was performed using the MapMan (Rasmusson *et al.* 2009) software tool. Changes in gene expression in *dapat* plants are further provided in Supplementary Tables S1, S2 at *JXB* online. Microarrays data were deposited at ArrayExpress (https://www.ebi.ac.uk/arrayexpress/) as accession number E-MTAB-5315.

### Gene expression analysis

Quantitative real-time PCR (qPCR) analysis was performed with three biological replicates, using gene-specific qPCR oligonucleotide pairs, designed with the Primer Express software (Applied Biosystems). *Ubiquitin C* (At5g25760) was used as the internal standard. The sequences of the specific oligonucleotides are provided in Supplementary Table S3. DNase-treated total RNA was reverse-transcribed using AMV Reverse Transcriptase (EurX Ltd) at a final concentration of 50 ng μl^−1^. Reactions were performed using the StepOnePlus™ Real-Time PCR System (Applied Biosystems) and amplifications were performed using the SYBR Green PCR Master Mix (Applied Biosystems). The relative levels of mRNAs were determined using the 2^−ΔΔ*C*t^ method (Livak and Schmittgen, 2001). The fold change data are presented as the ratio *dapat*/WT.

### Metabolite profiling

Metabolite profiling was performed based on the established gas chromatography–mass spectrometry (GC-MS) protocol of Lisec *et al.* (2006). Both chromatograms and mass spectra were evaluated using TAGFINDER software (Luedemann *et al.*, 2008). Metabolites were identified in comparison with database entries of authentic standards (Kopka *et al.*, 2005; Schauer *et al.*, 2005). Identification and annotation of detected peaks followed the recommendations for reporting metabolite data described in Fernie *et al.* (2011) and the full dataset is shown in Supplementary Table S4.

### 2D gel electrophoresis

Total leaf proteins were homogenized in 900 µl of ice-cold extraction buffer (50 mM Tris–HCl pH 8.5, 5 mM EDTA, 100 mM KCl, 1% (w/v) 1,4-dithiothreitol (DTT), 30% (w/v) sucrose, 2% phenylmethylsulfonyl fluoride) and vortexed for 30 s. The supernatant was recovered into a new tube and added to 900 µl of ice-cold Tris-buffered phenol (pH 8.0) and vortexed for 15 min at 4 °C followed by centrifugation (3 min, 6000 *g*, 4 °C). The phenolic phase was recovered into a new tube and re-extracted with 900 µl of ice-cold extraction buffer and vortexed for 30 s followed by centrifugation (3 min, 6000 *g*, 4 °C). The phenolic phase was further collected and precipitated overnight with 1 ml of 100 mM methanol–ammonium acetate at −20°C. After precipitation, the pellet was centrifuged (30 min, 16000 *g*, 4 °C) and rinsed with ice-cold acetone–DTT (0.2%) at −20 °C for 1 h. The sample was air-dried and resuspended in lysis buffer (7 M urea, 2 M thiourea, 4% CHAPS, 0.8% IPG-buffer (Amersham Biosciences), 1% DTT). Protein concentration was determined by the Bradford reagent (Bradford, 1976). An 850 µg aliquot of protein was diluted with a rehydration buffer (7 M urea, 2 M thiourea, 0.5% CHAPS, 10% glycerol, 0.002% bromophenol blue, 0.5% IPG-buffer) and loaded in strips of 18 cm, pH 4–7 linear for 16 h. Isoelectric focalization (IEF) was carried out in IPGphor at 20 °C with 50 µA per strip in the following conditions: 12 h at 200 V (step), 1 hour at 500 V (step), 600 V h at 1000 V (gradient), 13500 V h at 8000 V (gradient) and 18200 V h at 8000 V (step). After IEF, strips were equilibrated for 15 min on equilibrium buffer (6 M urea, 30% glycerol, 2% SDS, 0.002% bromophenol blue, 50 mM Tris pH 8.8) containing 1% DTT. Immediately, strips were equilibrated for 15 min on equilibrium buffer containing 4.5% iodoacetamide. The 2D electrophoresis was carried out at 15 °C in 12.5% polyacrylamide gel using the DaltSix System with the following conditions: 20 mA per strip for 30 min, 40 mA per strip for 6 h. The gel was fixed overnight and stained for 2 d in Coomassie Blue G-250 solution. Image acquisition was performed using an ImageScanner III (GE Healthcare) and images were analysed using ImageMaster 2D Platinum v. 7 software (GE Healthcare). Spot proteins that differed on ANOVA (*P*<0.05) were excised from the gel for trypsin digestion according to Shevchenko *et al.* (2006).

### Matrix-assisted laser desorption/ionization time-of-flight/time-of-flight mass spectrometry analysis

Trypsin digested proteins were concentrated and desalted using a hydrophobic C18 column (Millipore) then further analysed by matrix-assisted laser desorption/ionization time-of-flight/time-of-flight mass spectrometry (MALDI-TOF/TOF MS) using an AB SCIEX TOF/TOF 4800 proteomics analyzer (Applied Biosystems, USA) with an α-cyano-4-hydroxycinnamic acid (Sigma-Aldrich) matrix. Protein identification was based on peptide mass fingerprint and MS/MS ion search. The peak list obtained was analysed using a local Mascot 2.2.07 against the Uniprot_Arabidopsis_20140909 database considering a precursor tolerance of 0.1 Da for the product ions, allowing for deamination of asparagine and glutamine, methionine oxidation as a variable modification, carbamidomethylation as a fixed modification, two missed cleavages, and trypsin as the enzyme. The peptide and protein identification were statistically evaluated and validated at 90% probability using the Scaffold package (Proteome Software, Inc., Portland, OR, USA).

### Statistical analysis

The experimental design was completely randomized. Data were submitted to two-way analysis of variance (ANOVA) and tested for significant (*P<*0.05) differences using Student’s *t* test. All the statistical analyses were performed using an algorithm embedded in Microsoft Excel. In order to reduce the dimensionality of the metabolic dataset and identify the variables that explained a higher proportion of the total variance between the genotypes used here, a multivariate partial least-squares discriminant analysis (PLS-DA) (principal component analysis; PCA) with all metabolite data were used with the Excel add-in Multibase package (Numeral Dynamics, Japan).

## Results

### The DAPAT mutation reduces plant growth

**Fig. 2.**
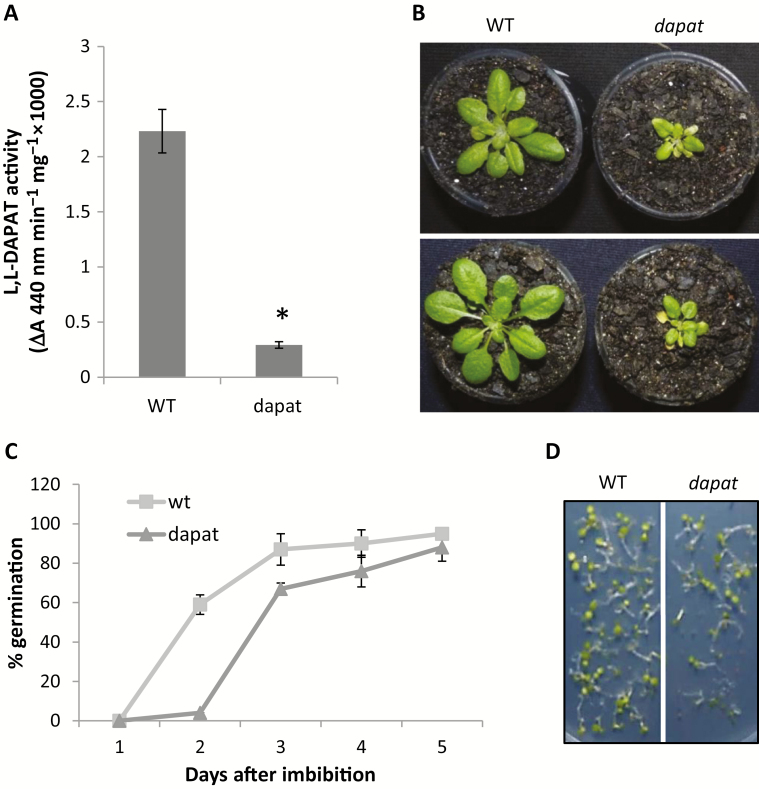
Effects of lysine biosynthesis deficiency observed in *dapat* mutant plants. (A) L,L-Diaminopimelate aminotransferase (DAPAT) enzyme activity showing drastic reduction in mutant plants. (B) Arabidopsis plants grown in short day conditions as described in ‘Materials and methods’. (C) WT and *dapat* seed germination assay showing a delay in germination of *dapat* seeds. (D) Arabidopsis seedling establishment of WT and *dapat* 5 d after start of germination. While WT showed germination of all seeds, a few *dapat* seeds were unable to germinate.

To investigate the contribution of impaired Lys biosynthesis on growth and metabolism, we have used an Arabidopsis mutant with reduced activity of the Lys biosynthesis enzyme DAPAT. Since *DAPAT* knockout plants are embryo lethal and this phenotype cannot be rescued by chemical application of Lys (Dobson *et al.*, 2011), we concentrated our analysis on a single mutation in the LL-DAPAT gene resulting in an impairment of enzyme activity. Lower DAPAT activity was determined showing that the *dapat* mutant has only 10% of total DAPAT activity observed in WT plants (Fig. 2A). In good agreement with previous results (Song *et al.*, 2004), *dapat* plants showed a clear decrease of rosette diameter (Fig. 2B; Table 1). This was coupled to a lower total number of leaves, as well as a strong decrease in both fresh and dry weight accumulation (Table 1). To study in detail the impact of the DAPAT mutation on plant growth we next compared the development of WT and *dapat* plants. Germination kinetics displayed a clear delay in *dapat* mutant plants (Fig. 2C) whilst a small number of *dapat* seeds were incapable of germination (Fig. 2D).

**Table 1.** Growth parameters observed in *dapat* plants

Parameter	WT	*dapat*
Rosette diameter (mm)	41.9 ± 1.2	**18.8 ± 1.3**
Number of leaves	17.5 ± 0.5	**12.5 ± 0.3**
Rosette fresh weight (mg)	0.1203 ± 0.08	**0.0403 ± 0.01**
Rosette dry weight (mg)	0.0123 ± 0.01	**0.0048 ± 0.01**

Plants deficient in lysine biosynthesis showed a reduced aerial biomass with respect to the wild-type (WT) during vegetative growth (4 weeks old). Values are presented as mean ±SE of at least 13 independent biological replicates per genotype; bold indicates values that were determined by Student’s *t*-test to be significantly different (*P*<0.05) from the WT.

**Fig. 3.**
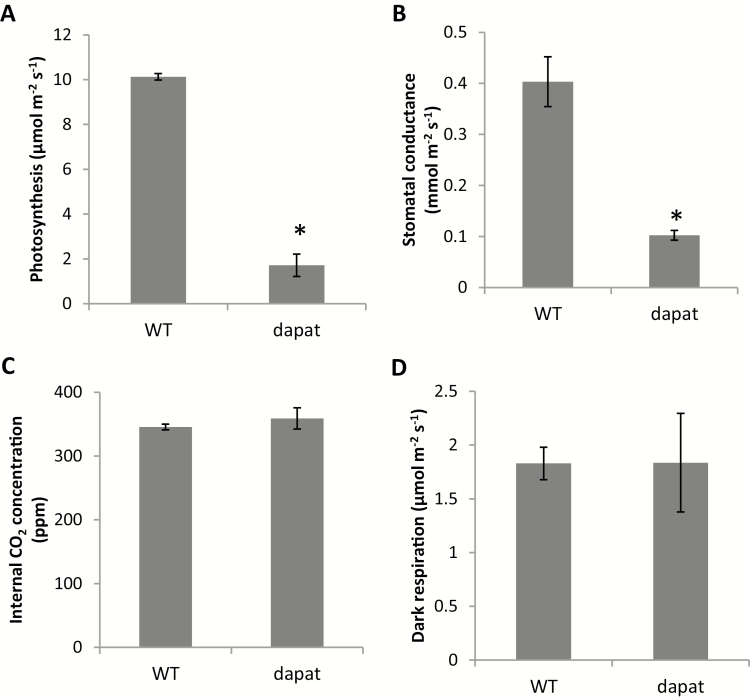
Changes in gas-exchange measurements in wild-type (WT) and mutant (*dapat*). (A) Photosynthesis. (B) Stomatal conductance to water vapor. (C) Internal CO_2_ concentration. (D) Dark respiration. Bars are means ±SE from five biological replicates; *significantly different (*P*<0.01) from WT by Student’s *t*-test.

Given that the DAPAT mutation resulted in impaired growth (Fig. 2B), we asked whether this phenomenon is associated with alterations in photosynthesis. We observed a significant reduction of both net photosynthesis (*A*) and stomatal conductance (*g*_s_) levels (83% and 75%, respectively) in relation to WT plants (Fig. 3A, B). By contrast, no significant difference in internal CO_2_ concentration (*C*_i_) was found between *dapat* and WT plants (Fig. 3C) indicating that decreased photosynthesis cannot be directly associated solely with stomatal limitations, but is most likely associated with biochemical limitations. Dark respiration (*R*_d_) was unaltered in *dapat* plants (Fig. 3D).

### Impact of DAPAT mutation on plant metabolism

**Fig. 4.**
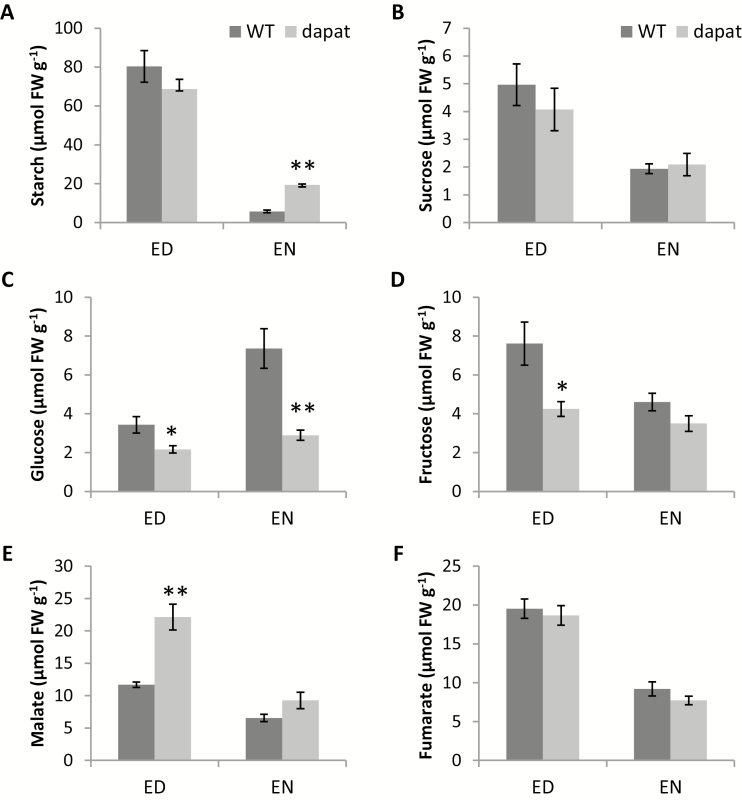
Effect of deficiency of lysine biosynthesis along diurnal cycle on starch (A), sucrose (B), glucose (C), fructose (D), malate (E), and fumarate (F) level. ED, end of day; EN, end of night. Values are means ±SE of five independent biological replicates. Asterisks designate values that were significantly different from WT (*P<0.05, **P<0.01) by Student’s *t*-test.

To further understand the phenotype observed in *dapat* plants, we next measured the levels of starch, soluble sugars, proteins, amino acids, and organic acids at the end of the day (ED) and at the end of the night (EN). Briefly, the levels of carbohydrates oscillated consistently, revealing one conspicuous feature that was clear when comparing the genotypes analysed here (Fig. 4). Sucrose was invariant between genotypes (Fig. 4B) whereas glucose and fructose (Fig. 4C, D, respectively) showed a similar pattern, with lower levels in *dapat* plants at both ED and EN when compared with WT plants. ANOVA was used to investigate statistical differences between genotypes and diurnal point (ED and EN). The mean separations were further compared by Student’s *t*-test and we observed similar statistical changes. There was a tendency for lower levels of starch in *dapat* plants at ED (Fig. 4A), in good agreement with the lower photosynthetic capacity (Fig. 3A). Interestingly, reduced starch consumption during the night was observed in *dapat* plants, and thus while about 7% of starch produced during the day was still present in WT plants, more than 20% of total starch synthesized remained at EN, suggesting impairment in starch degradation. Increased levels of malate were observed in *dapat* at ED compared with WT plants, reaching similar levels at EN (Fig. 4E). By contrast, no differences in the levels of fumarate were observed (Fig. 4F).

**Fig. 5.**
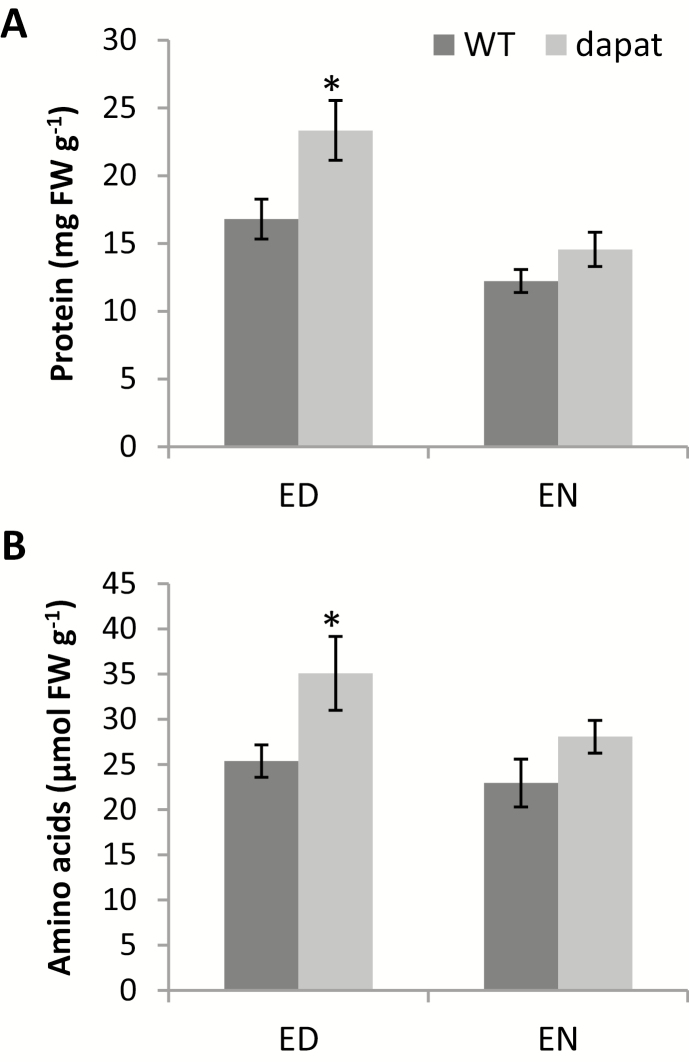
Effect of deficiency on lysine biosynthesis along a diurnal cycle on the total protein (A) and amino acid level (B). Values are means ±SE of five independent biological replicates. Asterisks designate values that were significantly different from WT (*P<0.05, **P<0.01) by Student’s *t*-test.

*dapat* plants contained higher levels of proteins (Fig. 5A) at ED with similar levels at EN suggesting that the consumption of protein during the night is higher in *dapat* plants (Fig. 5A). Indeed, when examining the decrease in protein levels between ED and EN, protein levels decreased more at EN in the *dapat* mutant (62.4% of levels observed at the ED) than WT (72.7%), revealing higher protein consumption during the night for *dapat* plants. Higher levels of free amino acids were observed at ED in *dapat* plants (Fig. 5B). However, determination of free amino acid levels at EN revealed that total free amino acids was similar between the *dapat* plants (with 80% residual amino acid content) and WT plants (with 72% residual content). These findings are supported by ANOVA showing significant difference (*P*<0.05) for major classes of metabolites (both protein and amino acids; Fig. 5); results of ANOVA and Student’s *t*-test were similar. Both statistical treatments reinforce our idea that nitrogen compounds (protein and total free amino acids, described in Fig. 5) are accumulated at ED and broken down during the night to further support energy metabolism as discussed below. Thus, changes observed suggested that an extensive reprogramming may be occurring in the *dapat* mutants, and as a consequence we extended our analysis to a broad transcriptional, proteomic, and metabolic analysis.

### Transcriptome changes induced by the DAPAT mutation

**Fig. 6.**
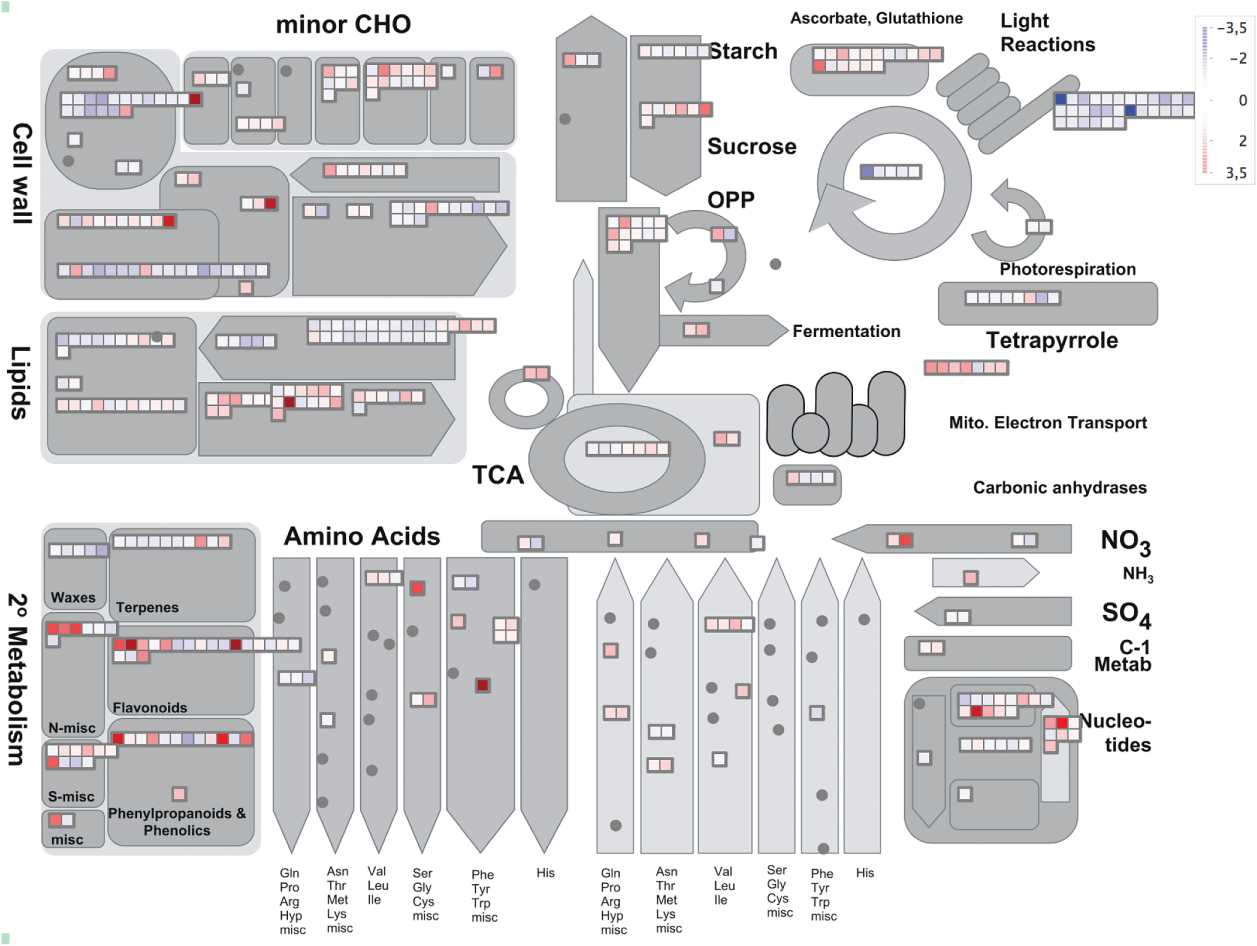
MapMan metabolic overview of the *dapat* mutant when compared with its wild-type correspondent. MapMan was loaded with 3138 transcripts that switched their expression at least 1.5-fold and showed ANOVA to *P*<0.05 into Arabidopsis major metabolic pathways. The log2 ratio values (*dapat*/WT) were plotted onto boxes in which up-regulated and down-regulated genes are indicated by the shading in accordance with the scale shown at the upper right.

To elucidate the effects of the DAPAT mutation on the global transcriptome, whole rosettes of 4-week-old-plants grown under short day conditions were harvested and their mRNAs subjected to an Affymetrix ATH1 microarray analysis. As our initial results indicated that the DAPAT mutation impacts both photosynthesis and carbohydrate turnover (Figs 3A, 4C) and are suggestive of higher protein degradation (Fig. 5A) most likely to support energy generation by alternative pathways of mitochondrial respiration, microarray analysis was carried out with samples harvested at EN. We focused on genes whose expression was significantly up- or down-regulated in the *dapat* mutant, compared with WT. It was found that the expression of 1982 and 1156 genes was significantly up- and down-regulated, respectively, in the *dapat* mutant compared with WT (Supplementary Tables S1, S2). These consistently up- and down-regulated genes were subjected to over-representation analysis using the tools embedded in the PageMan and MapMan software (Usadel *et al.*, 2006) and used to create an overview-of-metabolism diagram (Fig. 6; Supplementary Fig. S1). As would perhaps be expected, given that the *dapat* phenotype is due to a single amino acid substitution leading to a significant reduction of the LL-DAPAT activity (Hudson *et al.*, 2006), the expression of the LL-DAPAT (At4g33680) was not altered in our transcriptome profiling.

**Fig. 7.**
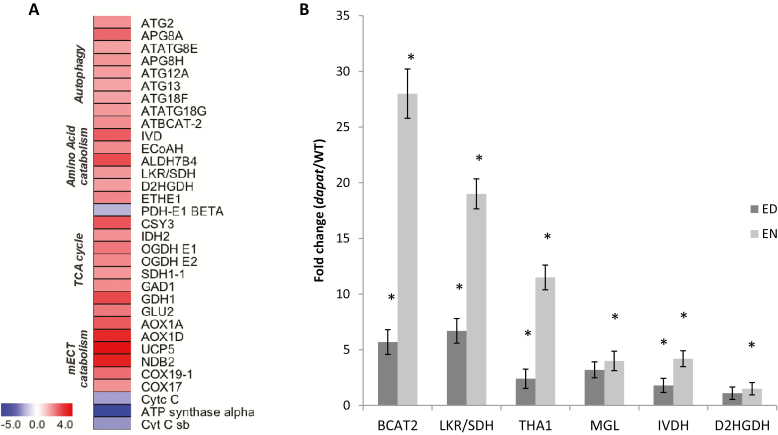
Gene expression in *dapat* mutant. (A) Heatmap of catabolic genes retrieved from *dapat* transcriptome. The log2 ratio (*dapat*/WT) values were plotted onto boxes in which up-regulated and down-regulated genes are indicated by shading in accordance with the scale shown at the lower left. (B) validation of trancriptome by quantitative PCR of amino acid catabolism genes along diurnal cycle. *Significantly different (*P*<0.05) from WT within each time point by Student’s *t*-test.

A range of stress-induced genes (BIN 20; 153 genes) were up-regulated, indicating that impaired Lys biosynthesis mimics a stressed plant phenotype. To further investigate the stress phenotype and accumulation of changes at the protein level in *dapat* plants, we performed a survey of genes related to the unfolded protein response (UPR) pathway, which could, at least partially, explain the dramatic visual phenotype. Notably, transcripts of UPR-related genes such as *IRE1*, *BiP* and transcription factors of the bZIP system were increased whereas the transcript levels of many genes of the UPR system were unaltered (see Supplementary Tables S1, S2). Therefore, caution must be shown in drawing any conclusions concerning the participation of the UPR system in determining the *dapat* phenotype. Furthermore, the transcriptome data suggested an early senescence phenotype in *dapat* plants. Accordingly, a group of transcription factors known as positive regulators of senescence were increased in *dapat* plants, such as *ORE1* (4.42-fold change), *ANAC29* (2.68-fold change), and *ANAC016* (2.62-fold change). Also, the WRKY transcription factor family was up-regulated, as shown by the overenrichment analysis by PageMan in which *WRKY45* increased 3.49-fold. Transcriptome data also revealed an increase of genes able to destabilize chloroplast integrity, such as *NYE1* and a recently identified *chloroplast vesiculation* (*CV*), which is involved in chloroplast degradation by an autophagy-independent pathway and senescence-associated vacuoles (SAVs) (Wang and Blumwald, 2014), presented here as unknown gene (Affymetrix ID 265913_at; Supplementary Table S1). Moreover, genes involved in protein degradation such as autophagy related genes (ATG) and *SAVs* showed a strong induction (Fig. 7A).

During the past years, it has been demonstrated that stress-induced senescence promotes amino acid catabolism supplying electrons to the mETC via the alternative pathway mediated by ETF–ETFQO (Araújo *et al.*, 2011*b*; Hildebrandt *et al.*, 2015). Hence, we next focused our analysis on amino acid metabolism and its association to the TCA cycle and ATP synthesis. Our transcriptome data showed a huge increase of genes encoding dehydrogenases associated to alternative respiration and amino acid catabolism, such as *Branched chain aminotransferase 2* (*BCAT2*), *IVDH*, and *Enoyl-CoA dehydrogenase* (*ECoAH*), all involved at BCAA degradation; *aldehyde dehydrogenase 7B4* (*ALDH7B4*), *Lys-ketoglutarate reductase/saccharopine dehydrogenase* (*LKR/SDH*), and *D2HGDH* (Lys degradation); *Persulfide dioxygenase* (*ETHE1*) (sulphur amino acid degradation); and *Glutamate decarboxylase* (*GAD1*), *Glutamate dehydrogenase* (*GDH1*), and *Fd-glutamate synthase* (*GLU2*) (glutamate degradation) (Fig. 7A). Simultaneously, isoforms of TCA cycle genes were clearly up-regulated (Fig. 7A). Notwithstanding, *PDH E1* beta subunit showed reduced expression. These data indicated that amino acid degradation may feed organic acids into the TCA cycle most likely by anaplerotic reactions (Araújo *et al.*, 2012). When considered together, our results suggest that Lys biosynthesis impairment caused by the DAPAT mutation leads to a molecular reprogramming that likely works by anticipating senescence in a putative stress situation leading to protein and amino acid degradation that ultimately support the activity of the oxidative phosphorylation system.

To validate our microarray data, we performed a qPCR analysis by selecting six genes related to amino acid catabolism: *BCAT2*, *LKR*/SDH, *Threonine aldolase* (*THA1*), *Methionine gamma-lyase* (*MGL*), *IVDH*, and *D2HGDH*. In addition, gene expression of these genes was performed at both ED and EN to evaluate their diurnal turnover. As shown in Fig. 7B, and in agreement with our transcriptomic analysis, the expression levels of all six genes were significantly higher in the *dapat* mutant, compared with WT, in both time points analysed and were also consistently higher at EN.

### The leaf proteome is affected by the DAPAT mutation

Since we have demonstrated that the DAPAT mutation has substantially altered levels of total protein and free amino acids (Fig. 5), we decided to carry out a robust proteome analysis via 2D electrophoresis (IEF/SDS-PAGE) in tandem with MALDI TOF/TOF (see Supplementary Fig. S2). We identified 95 proteins that changed their abundance in *dapat* plants at both ED and EN (Tables 2, 3; Supplementary Table S5). For simplicity, we first describe here the results obtained at ED and after, the results at EN. Importantly, the results are always presented in terms of changes that took place in the *dapat* plants in relation to WT.

**Table 2.** Identification of proteins with altered abundance in *dapat* with respect to the WT at the end of the day

Spot	Locus	Name	Fold	*P*-value
1	At2g45790	Phosphomanomutase	ND^*a*^	0.0021
2	At3g48870	HSP93-III	ND^*a*^	0.0244
3	AtCg00490	Rubisco large chain	7.1519	0.0228
4	At3g55800	Sedoheptulose-1,7-bisphosphatase	5.8696	0.0083
5	AtMg01190	ATP synthase subunit 1	4.0432	0.069
6	At5g13850	NACA3	1.7853	0.0459
7	At2g36880	*S*-Adenosylmethionine synthase 3	1.7631	0.0107
8	At1g57720	Elongation factor EF1B	1.7075	0.0397
9	At4g02520	Glutathione *S*-transferase 2	1.6004	0.0069
10	At1g42970	Glyceraldehyde-3-phosphate dehydrogenase subunit b	1.5973	0.0083
11	At3g01500	Beta carbonic anhydrase 1	1.5916	0.0193
12	At3g59970	Methylenetetrahydrofolate reductase 1	1.5300	0.0500
13	At4g16143	Importin α isoform 2	1.4309	0.0242
14	At1g09780	Phosphoglycerate mutase	1.3960	0.064
15	At5g25980	Glucoside glucohydrolase 2	1.3851	0.0445
16	At2g45140	Lipoxygenase 2	1.3793	0.060
17	At2g27720	60S acidic ribosomal protein	1.3676	0.0329
18	At4g04640	ATPase γ chloroplastic	1.3638	0.0288
19	At5g42020	BiP2	1.2800	0.0034
20	At1g23310	Ala:2-OG aminotransferase 1	1.2626	0.0125
21	At2g30860	Glutathione *S*-transferase 9	1.2419	0.0130
22	At3g11630	2-Cys peroxiredoxin	1.2416	0.0357
23	At2g47730	Glutathione *S*-transferase 8	1.1736	0.0016
24	At2g45140	Lipoxygenase 2	1.1517	0.0142
25	At1g20020	Ferredoxin–NADP reductase 2	1.1364	0.0031
26	At5g24490	30S ribosomal protein	0.8957	0.0130
27	At3g55440	Triose phosphate isomerase	0.8886	0.0690
28	At3g01500	Beta carbonic anhydrase 1	0.8596	0.0344
29	At4g25100	Fe-superoxide dismutase	0.8483	0.0112
30	At1g32060	Phosphoribulokinase	0.8449	0.0075
31	At3g01500	Beta carbonic anhydrase	0.8194	0.0032
32	At3g11630	2-Cys peroxiredoxin	0.7797	0.0574
33	At4g24280	HSP70	0.7579	0.0292
34	At3g09440	HSP70 protein 3	0.7367	0.0163
35	At1g01090	Pyruvate dehydrogenase E1α	0.7242	0.0592
36	At3g01500	Beta carbonic anhydrase 1	0.7216	0.0293
37	At3g50820	Oxygen envolving complex	0.7202	0.0355
38	At3g15360	Thioredoxin M-type 4	0.7151	0.0401
39	At5g39570	Uncharacterized protein	0.6994	0.0310
40	At5g25980	Beta glucosidase 37	0.6862	0.0012
41	At4g01850	S-Adenosylmethionine synthase 2	0.6586	0.0493
42	At2g39730	Rubisco activase	0.6551	0.0580
43	At1g68010	Hydroxypyruvate reductase 1	0.6392	0.0456
44	At2g38230	Pyridoxine biosynthesis 1	0.6208	0.0107
45	At5g06290	2-Cys peroxiredoxin	0.5735	0.0547
46	At3g63540	Thylakoid lumenal 19 kDa	0.5616	0.0337
47	At5g53490	Thylakoid lumenal 17.4 kDa	0.5429	0.0195
48	At1g54270	eIF4A	0.5140	0.0441
49	At5g52310	Responsive to dessication 29A	0.5124	0.0197
50	At5g39570	Uncharacterized protein	0.3539	0.0469
51	At5g17920	Methionine synthesis 1	0.3509	0.0051
52	At3g52960	Thioredoxin superfamily protein	0.0886	0.0030
53	At1g31180	Isopropylmalate dehydrogenase 3	ND^*b*^	<0.001
54	At1g78330	Glutathione *S*-transferase 19	ND^*b*^	<0.001
55	At3g50820	Oxygen envolving complex	ND^*b*^	<0.001
56	At1g53850	20S Proteasome α subinit E1	ND^*b*^	0.0069
57	At2g34430	LCB-II	ND^*b*^	0.0072
58	At3g26650	Glyceraldehyde-3-phosphate dehydrogenase subunit a	ND^*b*^	0.0252
59	At1g67090	Rubisco small chain	ND^*b*^	0.0254
60	At2g34430	LCB-II	ND^*b*^	0.0396

Proteins were separated by IEF/SDS-PAGE and spots were analysed by MALDI TOF/TOF (*n*=3). *P*<0.05.

^*a*^ ND: not detected in wild-type (Col-0).

^*b*^ ND: not detected in *dapat* mutant.

**Table 3.** Identification of proteins with altered abundance in *dapat* with respect to the WT at the end of the night

Spot	Locus	Name	Fold	*P*-value
61	At1g32470	GDC subunit H	ND^*a*^	<0.001
62	At5g63400	Adenylate kinase 4	ND^*a*^	0.0027
63	At4g38970	Fructose-bisphosphate aldolase 2	ND^*a*^	<0.001
64	At5g14200	3-Isopropylmalate dehydrogenase 1	ND^*a*^	<0.001
65	At1g51980	Mitochondrial-processing peptidase subunit α-1	ND^*a*^	<0.001
66	At5g11670	NADP-dependent malic enzyme 2	ND^*a*^	<0.001
67	At3g45140	Lipoxygenase 2, chloroplastic	ND^*a*^	<0.001
68	At1g29930	LCBll-b	ND^*a*^	<0.001
69	AtCg00490	Rubisco large chain	3.7305	<0.001
70	At4g13930	SHMT 4	3.1073	0.0021
71	At4g04640	ATP synthase γ chain 1.	3.0485	0.0001
72	AtCg00490	Rubisco large chain	2.111	0.0021
73	At5g14780	Formate dehydrogenase.	1.796	0.0006
74	At3g45140	Lipoxygenase 2	1.7279	0.0016
75	At5g39570	Uncharacterized protein	1.6958	0.0002
76	At5g37600	Glutamine synthetase isozyme 1	1.6922	0.0001
77	At4g02520	Glutathione *S*-transferase F2	1.5208	0.0005
78	At2g44350	Citrate synthase 4	1.36	9.91 × 10^−6^
79	At3g48870	Chaperone protein ClpC2	1.2211	0.0003
80	At1g32470	GDC subunit H	1.1909	0.0018
81	At2g33210	Chaperonin CPN60-like 1	1.1839	3.25 × 10^−5^
82	At1g21750	Protein disulfide isomerase-like 1-1	1.17	5.31 × 10^−6^
83	At1g20020	Ferredoxin–NADP reductase, leaf isozyme 2	1.1479	0.0005
84	At2g37660	Uncharacterized protein	0.8234	0.0010
85	At5g43940	Alcohol dehydrogenase 3	0.7418	0.0003
86	At5g27380	Glutathione synthetase	0.7312	1.58 × 10^−5^
87	At5g24780	Vegetative storage protein 1	0.5834	0.0001
88	At1g02930	Glutathione *S*-transferase F6	0.5808	2.06 × 10^−5^
89	At5g38420	Rubisco small 2B, chloroplastic	0.5295	0.0022
90	At1g42970	Glyceraldehyde-3-phosphate dehydrogenase, chloroplastic	0.4115	0.0010
91	AtCg00490	Rubisco large chain	0.3387	0.0010
92	At4g02520	Glutathione *S*-transferase F2	ND^*b*^	<0.001
93	At5g66190	Ferredoxin–NADP reductase isozyme 1	ND^*b*^	<0.001
94	At5g27380	Glutathione synthetase	ND^*b*^	<0.001
95	At1g32470	GDC subunit H	ND^*a*^	<0.001

Proteins were separated by IEF/SDS-PAGE and spots were analysed by MALDI TOF/TOF (*n*=3). *P*<0.05.

^*a*^ ND: not detected in wild-type (Col-0).

^*b*^ ND: not detected in *dapat* mutant.

By analysing proteins at ED we were able to identify several proteins related to photosynthesis (involved in both light and Calvin–Benson cycle reactions) and one protein related to photorespiration that significantly changed their abundance. Thus, although proteins related to photosystem II such as LHCb-II and oxygen evolving complex (subunit 33 kDa) decreased their abundance, proteins such as ferredoxin–NADP oxidoreductase 2 (At1g20020), located on PSI, and ATP synthase γ chain 1 (At4g04640) increased their abundance at ED in *dapat* plants. Interestingly, enzymes of the Calvin–Benson cycle were also affected in *dapat* mutant plants. In this vein, we observed that Rubisco large chain and seduheptulose 1,7-biphosphatase increased (7.15- and 5.8-fold, respectively) whereas Rubisco small chain 1a, phosphoribulokinase (PRK), glyceraldehyde 3-phosphate dehydrogenase (GAPDH) and Rubisco activase reduced their abundance (Table 2). In addition, redox-related proteins involved in the regulation of photosynthesis and others chloroplastic reactions such as thioredoxin m4, 2-Cys peroxidase A- and B-type, thioredoxin superfamily protein and plenty of gluthatione *S*-transferase isoforms (e.g. GSF2, GSF8, GFS9, and GSF18) were also identified and most of them were down-regulated. Regarding enzymes involved in photorespiration, only hydroxypyruvate reductase 1 was significantly reduced in *dapat* plants.

Our proteomic approach also provided some insights into amino acid metabolism at ED. We found an increase in glutamine:2-oxoglutarate aminotransferase (GOGAT) and also in proteins related to hormone metabolism such as *S*-adenosylmethionine synthase 2 and 3, which decreased and increased, respectively (Table 2). Lipoxigenase 2, which is related to jasmonate metabolism, also increased at ED.

When analysing proteome changes at EN, we observed the presence of 34 spots corresponding to 29 proteins differentially abundant in *dapat* plants (Table 3). Interestingly, most of those proteins, which accumulated in *dapat* plants, are related to energy metabolism and particularly located in the mitochondria [e.g glycine decarboxylase (GDC), serine hydroxymetyl transferase (SHMT) and citrate synthase (CS4); Table 3]. Accordingly, up-regulation of peptidase and chaperon CPN60-like 1, which are related to import and folding of novel mitochondrial proteins, demonstrates that mitochondrial metabolism is more active and that it is likely able to play a pivotal function concerning metabolic reprogramming in *dapat* plants. We also observed that CS4 was the only up-regulated protein belonging to the TCA cycle. In addition, fructose-bisphosphate aldolase 2 (FBPK2) and cytosolic malic enzyme 2 (cME2) increased at EN, suggesting an augmentation of the energetic metabolism in *dapat* plants. In agreement, the increased levels of proteins related to NADH production such as GDC subunit H, SHMT4, and formate dehydrogenase suggest that metabolic reprogramming is occurring in *dapat* plants most likely to use alternative energy sources such as amino acids. Furthermore, the decreased levels of alcohol dehydrogenase 3 may suggest a reduced glycolysis in *dapat* at night, giving further support to the contention that a disorder of carbon breakdown is most likely taking place in this mutant.

### Metabolome analysis reveals a metabolic reprograming in the *dapat* mutant

**Fig. 8.**
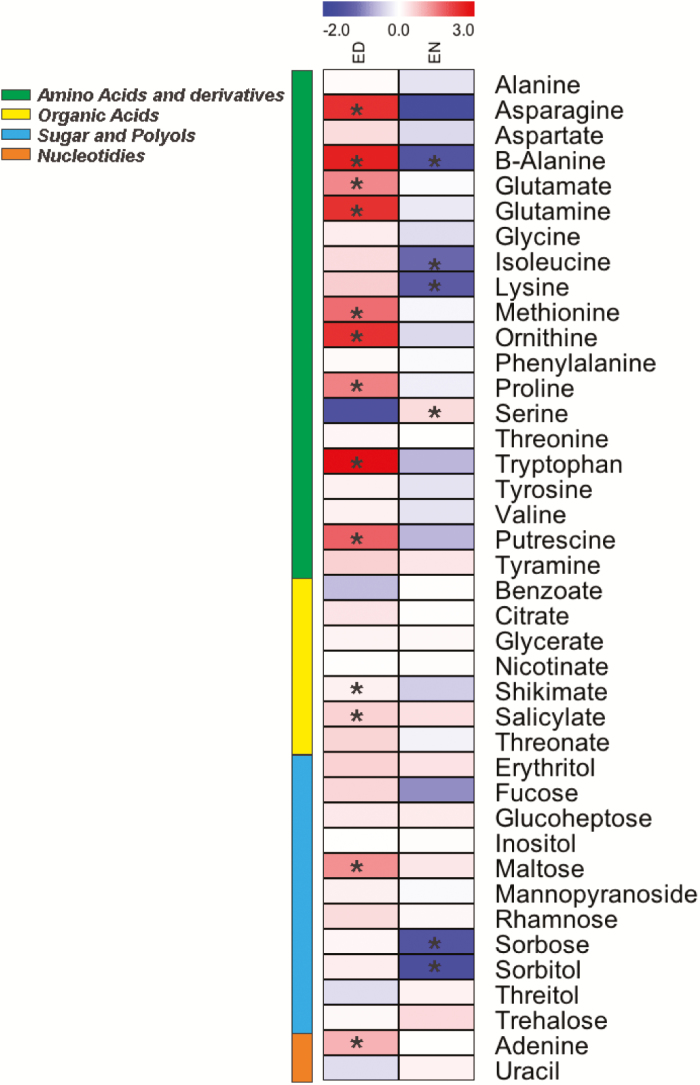
Metabolic profiling of *dapat* mutant along diurnal cycle. ED, end of day; EN, end of night. Metabolites were determined as described in ‘Materials and methods’. The full datasets from these metabolic profiling studies are additionally available in Supplementary Table S4. The colour code of the heat map is given as the log_2_ scale. Data are normalized with respect to the mean response calculated for WT (to allow statistical assessment, individual plants from this set were normalized in the same way). Values are means ±SE of four independent biological replicates. *Significantly different from WT (*P*<0.05) by Student’s *t*-test.

To explore the consequences of the DAPAT mutation on the major primary pathways of plant metabolism, an established GC-MS protocol (Lisec *et al.*, 2006) was used. The data obtained are displayed in a heat map (Fig. 8) in order to provide an easy overview (the full dataset is provided in Supplementary Table S4). From this display, it is noticeable that there were considerable changes in the levels of metabolites at both ED and EN in the *dapat* mutant. The levels of Lys in *dapat* plants at ED did not change, although surprisingly, a strong reduction was observed at EN for this amino acid. Furthermore, the levels of shikimic acid and salicylic acid were higher in *dapat* plants (Fig. 8). It is important to mention that the increased levels of salicylic acid were observed even when this mutant grew without pathogen attack or other stress conditions (Rate and Greenberg, 2001). The reduced DAPAT activity resulted in an accumulation of metabolites at ED known to be related to stress response such as proline and β-alanine (2.75- and 6.09-fold, respectively). In addition, putrescine, another stress-related metabolite (Wulff-Zottele *et al.*, 2010), followed the same behaviour with increased levels (3.45-fold) at ED. Furthermore, ornithine, which increased 5.17-fold at ED, is a central metabolite involved in the biosynthesis of polyamines (Majumdar *et al.*, 2013) and proline. Taken together, these findings suggest that the DAPAT mutation may result in a putative stress situation even when the plants are growing under optimal conditions.

The aspartate-family amino acids aspartate, methionine, and isoleucine accumulated significantly at ED in *dapat* plants. In addition, isoleucine decreased at EN, similarly to the situation observed for Lys. Perhaps one of the most interesting metabolic changes was that of tryptophan, which increased more than 8-fold at ED, but was reduced to 0.65 (relative level) at EN. Our metabolic profile also showed that the DAPAT mutation culminated in increased levels of glutamine and glutamate at ED (5.13- and 2.66-fold, respectively). Interestingly, serine and glycine showed an opposite behaviour and thus at ED glycine showed increased levels while serine decreased. In turn, at EN glycine had reduced levels whereas serine accumulated. Given the major changes in amino acid content of *dapat* plants, the influence of each amino acid in the total amino acid pool of *dapat* plants compared with its respective WT is provided as Supplementary Fig. S3. The amino acid pool of *dapat* is over-represented by several amino acids including tryptophan, β-alanine, asparagine, and ornithine at ED. By contrast, at EN the amino acid pool is relatively more balanced in *dapat* plants. Thus, the major changes caused by the DAPAT mutation occur at the end of the light period.

### Related metabolic responses among *dapat*, *dhdps2*, *kin10*, and *lht* mutants

To address whether the metabolite changes are triggered specifically by changes in Lys metabolism caused by the DAPAT mutation, we analysed metabolic responses of other mutants involved in (i) amino acid metabolism (*dhdps2*), (ii) amino acid transport (*lht1-1*), and (iii) stress signalling (*kin10*) (see Supplementary Tables S6, S7). *dhdps*-2 mutants, with impairments in Lys biosynthesis via the disruption of another step of the pathway, have also been characterized by growth rate reductions, affecting both leaves and roots (Sarrobert *et al.*, 2000). Growth reductions coupled with changes in plant architecture and lower yield have also been reported in double mutants of *dhps-1* and *dhps-2* (Jones-Held *et al.*, 2012). In addition, lines carrying insertions in *LHT1* showed reduced growth compared with the WT (Hirner *et al.*, 2006), whilst *KIN10* overexpression mutants displayed reduction of shoots and roots under normal growth conditions (Baena-González *et al.*, 2007). We therefore selected mutant plants that have a similar growth phenotype to *dapat* mutants. In order to obtain further insight into the metabolic changes that were unequivocally caused by the DAPAT mutation, we first focused on Lys biosynthesis by comparing *dapat* and plants lacking dihydrodipicolinate synthase (DHDPS). DHPDS is the first enzyme of the Lys biosynthesis pathway, and the *DHDPS2* gene (At2g45440) encodes one of two DHDPS isozymes that contributes to the majority of the total DHDPS activity in Arabidopsis. T‐DNA insertion lines of DHDPS2 display relatively lower Lys biosynthesis, and, as a result of this, a strongly enhanced synthesis of threonine (Craciun *et al.*, 2000). Analysis of the metabolic profile displayed significant differences between *dapat* and *dhdps2* mutants (Supplementary Fig. S4;Supplementary Table S6). The significantly increased levels of amino acids in *dapat* were, however, not observed in *dhps2* mutants. In fact, changes in amino acid composition have been previously observed mainly in the root tissue of *dhps2* mutants (Sarrobert *et al.*, 2000). In addition, higher levels of tryptophan were observed in *dapat* mutants, whereas in *dhps2* mutants the levels of this amino acid were invariant. During adverse conditions the accumulation of aromatic amino acids (e.g. tryptophan and phenylalanine) suggest their potential roles either as metabolic precursors or under conditions of stress (Galili *et al.*, 2016). Indeed increased levels of stress-related metabolites such as putrescine and proline were only observed in *dapat* mutants. In addition, the BCAAs and aromatic amino acids followed the same behaviour with higher levels only in *dapat* mutants.

Previous studies showed that modulation of biosynthesis and catabolic fluxes in the aspartate family pathway have a substantial influence on the TCA cycle (Angelovici *et al.*, 2009, 2011; Araújo *et al.*, 2010; Less *et al.*, 2011). Therefore, to confirm that the metabolic changes of *dapat* mutants are not due to a general response of amino acid and energetic metabolism modifications, we also analysed the metabolic profiles of *kin10* and *lht1-1*mutants. KIN10 is a subunit of the Arabidopsis SRnk1 that acts as a sensor for energy depletion (Baena-González *et al.*, 2007), and LHT1 (LYS HISTIDINE TRANSPORTER1) is a high affinity glutamine transporter that regulates the flux of central carbon/nitrogen metabolism into the Asp-family pathway (Hirner *et al.*, 2006). The full data obtained with metabolic profiling are provided (see Supplementary Fig. S4), whilst PCA was used to find differences between the profiles of *dapat*, *kin10*, and *lht-1*. The major variance of the dataset is covered by the first two principal components (PC1 covers 25% of the total variance and PC2 22%), which led to clear discrimination of *dapat* from the other mutants investigated (Supplementary Fig. S4; Supplementary Table S8). Although a detailed analysis of the entire data revealed no significant differences in sugars and organic acids between these genotypes, noticeable changes were found for amino acids (Supplementary Fig. S5B, C). The levels of isoleucine, valine, Lys, and tyrosine are significantly higher only in *kin10* and *lht-1* mutants. The higher levels of these amino acids have been associated to energy stress situations and they play key role in energy generation via the ETF–ETFQO system or by feeding TCA cycle intermediates (Araújo *et al.*, 2010; Barros *et al.*, 2017; Hirota *et al.*, 2018). Despite the metabolic pattern of *dapat*, *kin10*, and *lht-1* having a certain similarity, most amino acids increased only in *dapat* (Supplementary Fig. S5). Among them, glutamate, glutamine, and asparagine, precursors of the aspartate amino acid family, increased significantly more in *dapat* mutant plants suggesting that LL-DAPAT disruption leads to a specific readjustment of amino acid metabolism. Altogether, these results highlight that more pronounced changes in amino acid biosynthetic and catabolic pathways occur in *dapat* mutants, and they are in good agreement with our suggestion that mutation in the LL-DAPAT gene results in a unique molecular reprogramming.

## Discussion

By using a mutant in the Lys biosynthesis pathway we provided evidence of the pivotal importance of Lys as depicted by the growth impairment and photosynthesis reduction observed. Our results also highlight a novel aspect of Lys metabolism showing that the DAPAT mutation culminated in physiological and metabolic changes. The differential turnover of total protein and free amino acids observed between the *dapat* mutant and WT along the diurnal cycle (Fig. 5) indicate that the significant reduction in DAPAT activity, occurring in the *dapat* mutant (Fig. 2), has a major impact on protein consumption during the night, as depicted by our proteomic approach (Table 3; Supplementary Fig. S2). Collectively, these results demonstrate a previously unrecognized role of DAPAT and additionally Lys biosynthesis by showing their importance in affecting growth and primary metabolism with direct impact on protein consumption.

Our data demonstrated that photosynthesis was negatively affected in *dapat* plants (Fig. 3A). The reduction in photosynthetic rates may explain, at least partially, the lower accumulation of starch at ED in *dapat* plants (Fig. 4A). Reduction in photosynthesis is regulated by either stomatal movements or biochemical parameters associated with photosystem components and Calvin–Benson cycle enzymes. Nonetheless, internal CO_2_ concentration was similar between *dapat* and WT plants, suggesting that reductions in CO_2_ assimilation are caused by biochemical impairments or adjustments. Our findings demonstrated a decrease in PSII proteins and an increase in both PSI protein and ATP synthase γ chain 1 (Table 2). This opposite behaviour may be indicative of the occurrence of cyclic electron flow allowing the maintenance of chloroplastic electron transfer to sustain ATP synthesis. This finding is further supported by a disorder of redox proteins that regulate photosynthesis and others chloroplastic reactions (see Supplementary Table S1, S2). Notably, a reduction of PSII proteins associated with the specific reduction of thioredoxin *m4*, as previously observed (Serrato *et al.*, 2013), may explain the cyclic electron flow taking place in the *dapat* mutant. Furthermore, the reduced levels of enzymes of the Calvin–Benson cycle such as GAPDH and PRK (Table 2) might culminate with a decreased flux through this cycle. Accordingly, PRK–GAPDH forms a complex mediated by the protein CP12, which is a regulatory step of the Calvin–Benson cycle (Marri *et al.*, 2005; Serrato *et al.*, 2013). Remarkably, when CP12 is in its oxidized form, this complex shows lower activity (Marri *et al.*, 2009). Moreover, the activity of the PRK–CP12–GAPDH complex is regulated by thioredoxin (Howard *et al.*, 2008; Marri *et al.*, 2009). Our findings demonstrated a decrease in the levels of proteins of the thioredoxin system and this regulation is most likely associated with impairments of PRK–CP12–GAPDH, which becomes inactive, consequently reducing the Calvin–Benson cycle flow. In this scenario, where there is a decrease of proteins of the Calvin–Benson cycle as well as Rubisco content and Rubisco activase (Table 2), the reduced power generated as NADPH, which is normally used for CO_2_ assimilation, might be redirected to other reactions. Here, we hypothesize that the NADPH produced by the chloroplastic electron chain is used by NADP-dependent malate dehydrogenase to produce malate. In good agreement, our organic acid measurements showed an accumulation of malate at ED (Fig. 4E). Accordingly, impairments in stomatal conductance have been associated with changes in organic acid metabolism as observed in both fumarase and succinate dehydrogenase antisense lines (Nunes-Nesi *et al.*, 2007; Araújo *et al.*, 2011*a*). Combining physiological and biochemical approaches, we provide evidence that the DAPAT mutation impacts malate levels, partially explaining the stomatal impairments in *dapat* plants; however, the precise mechanistic link between Lys biosynthesis and malate levels, particularly at the guard cell level, remains to be identified.

We demonstrated that the DAPAT mutation culminates with higher levels of starch at EN and decreased rate of starch breakdown during the night indicating that both accumulation and turnover of starch are altered in *dapat* plants (Fig. 4A). The reduced capability of *dapat* plants to degraded starch can lead to a putative starchless condition, which might culminate in reduced growth (Fig. 2B; Table 1). In good agreement with that, tight regulation between starch turnover and growth has been extensively demonstrated (Sulpice *et al.*, 2009; Andriotis *et al.*, 2012; Ragel *et al.*, 2013) and that fast or incomplete exhaustion of starch culminated in reduced growth (Stitt and Zeeman, 2012). It should be mentioned, however, that the augmentation of protein content during the light period may lead to an increment of energy cost to sustain amino acid and protein synthesis (Hachiya *et al.*, 2007; Piques *et al.*, 2009; Raven, 2012), which can represent a large source of ATP consumption, leading also to growth reduction (Fig. 2B). Thus, it can be assumed that *dapat* plants are most likely unable to efficiently degrade starch and this impairment may result in the growth arrest observed in *dapat* plants. We hypothesize that *dapat* plants use alternative substrates to sustain mitochondrial respiration and ATP synthesis, since *dapat* and WT plants presented similar dark respiration (Fig. 3D), provided by higher protein degradation to feed alternative respiration, as previously demonstrated (Araújo *et al.*, 2010; Izumi *et al.*, 2013; Avin-Wittenberg *et al.*, 2015).

The Lys pathway can be assumed to be a respiratory bypass associated with 2-oxoglutarate production that is able to feed the TCA cycle (Araújo *et al.*, 2010; Boex-Fontvieille *et al.*, 2013). Following this assumption, our data provided novel insights into amino acid turnover during the diurnal cycle (Fig. 8). The daily fluctuation of both glutamate and glutamine in *dapat* plants indicates a strong variation of glutamate content. Intriguingly, the pattern observed in this mutant is clearly different from previous reports in which glutamate normally suffered a small oscillation during the day (Stitt *et al.*, 2002; Gibon *et al.*, 2006). Thus, strong variations in glutamate, glutamine, and proline seem to be associated with reduced aminotransferase activity caused by the DAPAT mutation. It is noteworthy that changes in metabolites observed in *dapat* plants are rather different from the changes verified in other mutants displaying lack of or low aminotransferase activity such as branched-chain amino acid aminotransferase 3 (*bcaa3*), tyrosine aminotransferase (*tat*), and ornithine-δ-aminotransferase (*oat*) (Funck *et al.*, 2008; Knill *et al.*, 2008; Riewe *et al.*, 2012). When taken together with other Arabidopsis mutants involved in amino acid metabolism and/or related to stress (see Supplementary Fig. S4), our results suggest that the DAPAT gene plays a unique or specific role in cellular metabolism.

Changes in glutamate observed in *dapat* plants might be explained by the increase in *Alanine:2-oxoglutarate aminotransferase* (At1g23310) at ED. It is reasonable to assume, therefore, that to maintain basal levels of glutamine, *dapat* plants make use of glutamate as a precursor of glutamine, proline, and ornithine, which in turn increased at ED (Fig. 8). Alternatively, glutamate and their derivative amino acids decreased at EN. This amino acid reduction during the night suggests that they can be converted into 2-oxoglutarate that goes into the TCA cycle allowing the production of NADH and ATP. Furthermore, in the presence of proline there is an increased activity of both proline dehydrogenase and glutamate dehydrogenase to maintain the production of 2-oxoglutarate, which may be completely oxidized in the TCA cycle to support energy production (Schertl *et al.*, 2014). Thus, glutamate and its derivatives are likely energetic sources supplying organic acids in this mutant. In agreement with this, our transcriptome data indicate an increase in glutamate degradation by GAD1, which is connected to the bypass for glutamate degradation to feed the TCA cycle with succinate mediated by the GABA shunt (Michaeli and Fromm, 2015).

**Fig. 9.**
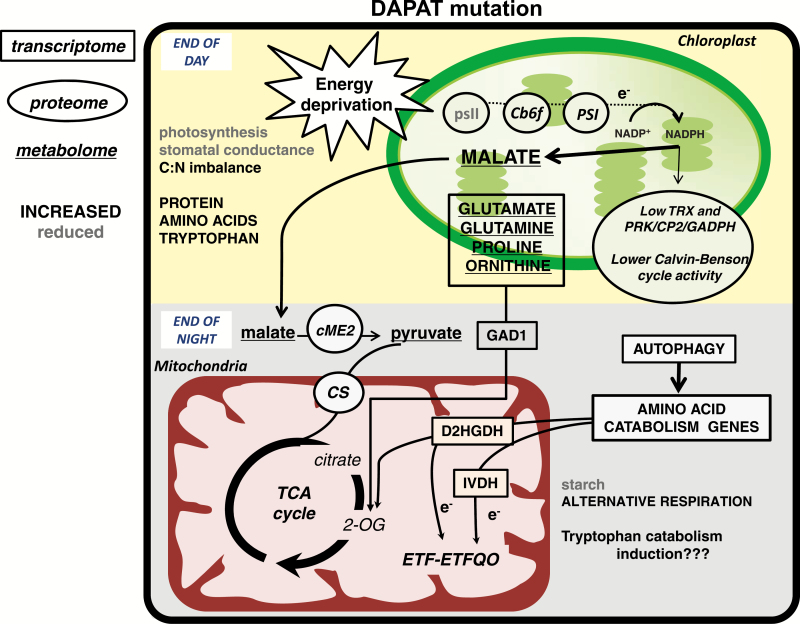
Schematic representation of metabolic reprogramming caused by the DAPAT mutation during diurnal cycle. Despite lower stomatal conductance, biochemical changes were responsible for lower photosynthesis, including an impact on carbohydrate metabolism resulting in putative energy deprivation and further accumulation of protein and amino acids, leading to C/N imbalance, which seems to be associated with the dwarfism phenotype in *dapat* plants. In order to obtain a metabolic adjustment, we postulate a hypothetical model in which malate is hyper-accumulated at the end of day (ED). Malate is further oxidized during the night by an alternative pathway associated with the cytosolic malic enzyme 2 to generated pyruvate and, thus, to sustain mitochondrial dark respiration at similar level when compared with WT plants. Furthermore, higher turnover of both protein and amino acids in *dapat* plants acts as a source for alternative respiration mediated by the ETF–ETFQO complex and dehydrogenases such as IVDH and D2HGDH. Data obtained from the transcriptome, proteome, and metabolome were integrated to build the mechanism described here. Rectangles represent transcript data; circles represent protein data, and underlined names represent metabolite data. Increased and reduced levels from the data obtained are shown in black uppercase and grey lowercase letters, respectively. Abbreviations: Cb6f, complex *b*_6_/*f*; CS, citrate synthase; cME2, cytosolic malic enzyme 2; D2HGDH, D-2-hydroxyglutarate dehydrogenase; DAPAT, L-L-diaminopimelate aminotransferase; ETF–ETFQO, electron-transfer flavoprotein–electron-transfer flavoprotein: ubiquinone oxidoredutase; GAD1, glutamate decarboxylase; GADPH, glyceraldehyde-3-phosphate dehydrogenase; IVDH, isovaleryl-CoA dehydrogenase; PRK, phosphoribulokinase; PSI, photosystem I; PSII, photosystem 2; TRX, thioredoxin; WT, wild type.

Protein degradation providing substrates to respiration is likely present in *dapat* mutants. Autophagy is induced in *dapat* plants (Fig. 7A), and recent studies have reported autophagy is able to provide alternative substrates for respiration (Avin-Wittenberg *et al.*, 2015; Barros *et al.*, 2017), in agreement with drastic reductions in both isoleucine and Lys as well as tryptophan at EN (Fig. 8). By integrating this metabolic data with the transcriptome, which displays increases of *IVDH* and *D2HGDH*, related to catabolism of BCAAs and Lys, respectively (Engqvist *et al.*, 2009; Araújo *et al.*, 2010; Peng *et al.*, 2015; Cavalcanti *et al.*, 2017), it is reasonable to assume that alternative respiratory pathways mediated by ETF–ETFQO are up-regulated in *dapat* plants. Furthermore, the maintenance of similar dark respiration rates in *dapat* plants might also occur through oxidation of malate that is accumulated during the light period. Diurnal oscillation in organic acid levels, especially malate, suggests that these organic acids are used as alternative respiratory substrates providing an important supply mainly under low carbohydrate conditions (Gibon *et al.*, 2009). In this vein, the increased levels of NADH-dependent cME2 coupled with simultaneous increases of citrate synthase, the first commissioned step of the TCA cycle, and the metabolite profiles described above allow us to postulate a pathway for the maintenance of respiration in situations where DAPAT mutation impairs plant growth. Moreover, differential metabolic behaviour between the distinct mutants used here (see Supplementary Figs S4, S5) is highly suggestive of a unique and specific metabolic reprograming in DAPAT mutant plants as summarized in Fig. 9. It thus seems reasonable to anticipate that the differences in growth and metabolism as depicted by changes in transcripts, proteins, and metabolites are most likely related to energetic factors occurring in response to the LL-DAPAT disruption. Our results thus suggest that the proper functioning of the pathway for Lys biosynthesis is important in fulfilling the energetic requirements to sustain plant growth and development.

We demonstrated here that impaired Lys biosynthesis caused by the DAPAT mutation culminated in constant stress conditions that resulted in an exquisite molecular and, consequently, physiological reprogramming. This reprogramming associated with photosynthetic reduction is in agreement with the dwarf phenotype (Fig. 2A) and that under stress conditions plants make use of alternative substrates for the maintenance of respiration (Araújo *et al.*, 2010, 2011*a*). Altogether, our results demonstrated that growth reduction observed in the *dapat* mutant is likely due to an imbalance in storage and breakdown of carbon and nitrogen sources uncoupling growth from primary metabolism. The specific mechanism in which DAPAT is metabolically involved is rather complicated (Fig. 9). Since Lys seems to be associated with feedback regulation at distinct levels (e.g. transcriptional or enzymatic), cross-pathway metabolic regulation associated with branching Lys metabolism or even other amino acids (Guyer *et al.*, 1995; Azevedo and Arruda, 2010; Ufaz and Galili, 2008) is usually observed. The complex cross-pathway regulation in which Lys metabolism might be inserted seems to be thus one major constraint on biotechnological approaches to enhance Lys in crops and cereal grains, which represents a major nutritional problem for humans and for feeding livestock in developing countries (Galili, 2011; Galili and Amir, 2013; Wang and Galili 2016). The optimization of Lys levels in plants thus requires a comprehensive understanding of the biological processes regulating the homeostasis of this essential amino acid as well as the metabolic consequences of this homeostasis.

In summary, our data provide compelling evidence that the DAPAT mutation leads to energy limitation and culminates with strong alterations in cellular metabolism, and that alternative substrates are used when there is an imbalance in Lys biosynthesis allowing the proper functioning of plant respiration and energy generation. Whilst these data provide a clear connection between mitochondrial metabolism and Lys biosynthesis, future investigation is still required including a deeper focus on growth connections to fully elucidate the precise factors underlying this metabolic phenotype that mimics stress conditions in *dapat* mutants.

## Supplementary data

Supplementary data are available at JXB online

Fig. S1. Bar chart of functional categories of differentially changed genes in *dapat* mutants (PageMan analysis; Usadel *et al.*, 2006).

Fig. S2. 2D gel maps of rosette of Arabidopsis at two points: (A) end of day and (B) end of night (ED)

Fig. S3. Pie chart comparing the impact of each amino acid on amino acid pools between *dapat* and wild-type along diurnal cycle: (A) at the end of day (ED) and (B) at the end of night (EN).

Fig. S4. Heatmap comparison of GC-MS metabolite profiling between relative values of *dapat* and *dhdps2* with the corresponding wild-type Col-0 and WS, respectively.

Fig. S5. Differences among *dapat*, *kin10* overexpression, and *lht1-1* mutants

Table S1. Up-regulated genes in *dapat* mutant transcriptome.

Table S2. Down-regulated genes in *dapat* mutant transcriptome.

Table S3. List of primers utilized for qPCR.

Table S4. Metabolite profiling in leaves of WT and *dapat* plants.

Table S5. Detailed proteomic data.

Table S6. Metabolite profiling in leaves of *dhdps2* and *dapat* as well as their corresponding wild-type Col-0 and WS, respectively.

Table S7. Metabolite profiling in leaves of *dapat*, *lht*, and *kin10* plants.

Table S8. PCA values.

Supplementary Tables S1-S8

Supplementary Figures S1-S5

## References

[CIT0001] Andriotis VM, Pike MJ, Schwarz SL, Rawsthorne S, Wang TL, Smith AM 2012 Altered starch turnover in the maternal plant has major effects on Arabidopsis fruit growth and seed composition. Plant Physiology 160, 1175–1186. 22942388 10.1104/pp.112.205062 PMC3490605

[CIT0002] Angelovici R, Fait A, Fernie AR, Galili G 2011 A seed high-lysine trait is negatively associated with the TCA cycle and slows down Arabidopsis seed germination. New Phytologist 189, 148–159. 20946418 10.1111/j.1469-8137.2010.03478.x

[CIT0003] Angelovici R, Fait A, Zhu X, Szymanski J, Feldmesser E, Fernie AR, Galili G 2009 Deciphering transcriptional and metabolic networks associated with lysine metabolism during Arabidopsis seed development. Plant Physiology 151, 2058–2072. 19783646 10.1104/pp.109.145631 PMC2785976

[CIT0004] Araújo WL, Ishizaki K, Nunes-Nesi A, et al 2010 Identification of the 2-hydroxyglutarate and isovaleryl-CoA dehydrogenases as alternative electron donors linking lysine catabolism to the electron transport chain of *Arabidopsis* mitochondria. The Plant Cell 22, 1549–1563. 20501910 10.1105/tpc.110.075630 PMC2899879

[CIT0005] Araújo WL, Nunes-Nesi A, Nikoloski Z, Sweetlove LJ, Fernie AR 2012 Metabolic control and regulation of the tricarboxylic acid cycle in photosynthetic and heterotrophic plant tissues. Plant, Cell & Environment 35, 1–21. 10.1111/j.1365-3040.2011.02332.x 21477125

[CIT0006] Araújo WL, Nunes-Nesi A, Osorio S, et al 2011a Antisense inhibition of the iron-sulphur subunit of succinate dehydrogenase enhances photosynthesis and growth in tomato via an organic acid-mediated effect on stomatal aperture. The Plant Cell 23, 600–627. 21307286 10.1105/tpc.110.081224 PMC3077794

[CIT0007] Araújo WL, Tohge T, Ishizaki K, Leaver CJ, Fernie AR 2011b Protein degradation – an alternative respiratory substrate for stressed plants. Trends in Plant Science 16, 489–498. 21684795 10.1016/j.tplants.2011.05.008

[CIT0008] Avin-Wittenberg T, Bajdzienko K, Wittenberg G, Alseekh S, Tohge T, Bock R, Giavalisco P, Fernie AR 2015 Global analysis of the role of autophagy in cellular metabolism and energy homeostasis in Arabidopsis seedlings under carbon starvation. The Plant Cell 27, 306–322. 25649436 10.1105/tpc.114.134205 PMC4456922

[CIT0009] Azevedo RA, Arruda P 2010 High-lysine maize: the key discoveries that have made it possible. Amino Acids 39, 979–989. 20373119 10.1007/s00726-010-0576-5

[CIT0010] Baena-González E, Rolland F, Thevelein JM, Sheen J 2007 A central integrator of transcription networks in plant stress and energy signalling. Nature 448, 938–942. 17671505 10.1038/nature06069

[CIT0011] Baena-González E, Sheen J 2008 Convergent energy and stress signaling. Trends in Plant Science 13, 474–482. 18701338 10.1016/j.tplants.2008.06.006 PMC3075853

[CIT0012] Barros JAS, Cavalcanti JHF, Medeiros DB, Nunes-Nesi A, Avin-Wittenberg T, Fernie AR, Araújo WL 2017 Autophagy deficiency compromises alternative pathways of respiration following energy deprivation in *Arabidopsis thaliana*. Plant Physiology 175, 62–76. 28710132 10.1104/pp.16.01576 PMC5580740

[CIT0013] Boex-Fontvieille ER, Gauthier PP, Gilard F, Hodges M, Tcherkez GG 2013 A new anaplerotic respiratory pathway involving lysine biosynthesis in isocitrate dehydrogenase-deficient *Arabidopsis* mutants. New Phytologist 199, 673–682. 23718121 10.1111/nph.12319

[CIT0014] Bouma TJ, Devisser R, Janssen J, Dekock MJ, Vanleeuwen PH, Lambers H 1994 Respiratory energy requirements and rate of protein turnover in vivo determined by the use of an inhibitor of protein synthesis and a probe to assess its effect. Physiologia Plantarum 92, 585–594.

[CIT0015] Bradford MM 1976 A rapid and sensitive method for the quantitation of microgram quantities of protein utilizing the principle of protein-dye binding. Analytical Biochemistry 72, 248–254. 942051 10.1016/0003-2697(76)90527-3

[CIT0016] Buchanan-Wollaston V, Page T, Harrison E, et al 2005 Comparative transcriptome analysis reveals significant differences in gene expression and signalling pathways between developmental and dark/starvation-induced senescence in Arabidopsis. The Plant Journal 42, 567–585. 15860015 10.1111/j.1365-313X.2005.02399.x

[CIT0017] Cavalcanti JHF, Quinhones CGS, Schertl P, Brito DS, Eubel H, Hildebrandt T, Nunes-Nesi A, Braun HP, Araújo WL 2017 Differential impact of amino acids on OXPHOS system activity following carbohydrate starvation in Arabidopsis cell suspensions. Physiologia Plantarum 161, 451–467. 28767134 10.1111/ppl.12612

[CIT0018] Clark TJ, Lu Y 2015 Analysis of loss-of-function mutants in aspartate kinase and homoserine dehydrogenase genes points to complexity in the regulation of aspartate-derived amino acid contents. Plant Physiology 168, 1512–1526. 26063505 10.1104/pp.15.00364 PMC4528744

[CIT0019] Craciun A, Jacobs M, Vauterin M 2000 Arabidopsis loss-of-function mutant in the lysine pathway points out complex regulation mechanisms. FEBS Letters 487, 234–238. 11150516 10.1016/s0014-5793(00)02303-6

[CIT0020] Dobson RC, Girón I, Hudson AO 2011 *L*,*L*-Diaminopimelate aminotransferase from *Chlamydomonas reinhardtii*: a target for algaecide development. PLoS ONE 6, e20439. 21633707 10.1371/journal.pone.0020439 PMC3102117

[CIT0021] Ebisuno T, Shigesada K, Katsuki H 1975 D-α-Hydroxyglutarate dehydrogenase of *Rhodospirillum rubrum*. Journal of Biochemistry 78, 1321–1329. 5424 10.1093/oxfordjournals.jbchem.a131030

[CIT0022] Engqvist M, Drincovich MF, Flügge UI, Maurino VG 2009 Two D-2-hydroxy-acid dehydrogenases in *Arabidopsis thaliana* with catalytic capacities to participate in the last reactions of the methylglyoxal and β-oxidation pathways. The Journal of Biological Chemistry 284, 25026–25037. 19586914 10.1074/jbc.M109.021253 PMC2757207

[CIT0023] Fernie AR, Aharoni A, Willmitzer L, Stitt M, Tohge T, Kopka J, Carroll AJ, Saito K, Fraser PD, DeLuca V 2011 Recommendations for reporting metabolite data. The Plant Cell 23, 2477–2482. 21771932 10.1105/tpc.111.086272 PMC3226225

[CIT0024] Fernie AR, Carrari F, Sweetlove LJ 2004 Respiratory metabolism: glycolysis, the TCA cycle and mitochondrial electron transport. Current Opinion in Plant Biology 7, 254–261. 15134745 10.1016/j.pbi.2004.03.007

[CIT0025] Fernie AR, Roscher A, Ratcliffe RG, Kruger NJ 2001 Fructose 2,6-bisphosphate activates pyrophosphate: fructose-6-phosphate 1-phosphotransferase and increases triose phosphate to hexose phosphate cycling in heterotrophic cells. Planta 212, 250–263. 11216846 10.1007/s004250000386

[CIT0026] Funck D, Stadelhofer B, Koch W 2008 Ornithine-δ-aminotransferase is essential for arginine catabolism but not for proline biosynthesis. BMC Plant Biology 17, 1–14. 10.1186/1471-2229-8-40 PMC2377265 18419821

[CIT0027] Galili G 2011 The aspartate-family pathway of plants: linking production of essential amino acids with energy and stress regulation. Plant Signaling and Behavior 6, 192–195. 21512320 10.4161/psb.6.2.14425 PMC3121977

[CIT0028] Galili G, Amir R 2013 Fortifying plants with the essential amino acids lysine and methionine to improve nutritional quality. Plant Biotechnology Journal 11, 211–222. 23279001 10.1111/pbi.12025

[CIT0029] Galili G, Amir R, Fernie AR 2016 The regulation of essential amino acid synthesis and accumulation in plants. Annual Review of Plant Biology 67, 153–178. 10.1146/annurev-arplant-043015-112213 26735064

[CIT0030] Galili G, Avin-Wittenberg T, Angelovici R, Fernie AR 2014 The role of photosynthesis and amino acid metabolism in the energy status during seed development. Frontiers in Plant Science 5, 447. 25232362 10.3389/fpls.2014.00447 PMC4153028

[CIT0031] Gibon Y, Pyl ET, Sulpice R, Lunn JE, Höhne M, Günther M, Stitt M 2009 Adjustment of growth, starch turnover, protein content and central metabolism to a decrease of the carbon supply when *Arabidopsis* is grown in very short photoperiods. Plant, Cell & Environment 32, 859–874. 10.1111/j.1365-3040.2009.01965.x 19236606

[CIT0032] Gibon Y, Usadel B, Blaesing OE, Kamlage B, Hoehne M, Trethewey R, Stitt M 2006 Integration of metabolite with transcript and enzyme activity profiling during diurnal cycles in *Arabidopsis* rosettes. Genome Biology 7, R76. 16916443 10.1186/gb-2006-7-8-r76 PMC1779593

[CIT0033] Gu L, Jones AD, Last RL 2010 Broad connections in the Arabidopsis seed metabolic network revealed by metabolite profiling of an amino acid catabolism mutant. The Plant Journal 61, 579–590. 19929878 10.1111/j.1365-313X.2009.04083.x

[CIT0034] Guyer D, Patton D, Ward E 1995 Evidence for cross-pathway regulation of metabolic gene expression in plants. Proceedings of the National Academy of Sciences, USA 23, 4997–5000. 10.1073/pnas.92.11.4997 PMC41834 7761437

[CIT0035] Hachiya T, Terashima I, Noguchi K 2007 Increase in respiratory cost at high growth temperature is attributed to high protein turnover cost in *Petunia* × *hybrida* petals. Plant, Cell & Environment 30, 1269–1283. 10.1111/j.1365-3040.2007.01701.x 17727417

[CIT0036] Hildebrandt TM, Nunes Nesi A, Araújo WL, Braun HP 2015 Amino acid catabolism in plants. Molecular Plant 8, 1563–1579. 26384576 10.1016/j.molp.2015.09.005

[CIT0037] Hirner A, Ladwig F, Stransky H, Okumoto S, Keinath M, Harms A, Frommer WB, Koch W 2006 Arabidopsis LHT1 is a high-affinity transporter for cellular amino acid uptake in both root epidermis and leaf mesophyll. The Plant Cell 18, 1931–1946. 16816136 10.1105/tpc.106.041012 PMC1533986

[CIT0038] Hirota T, Izumi M, Wada S, Makino A, Ishida H 2018 Vacuolar protein degradation via autophagy provides substrates to amino acid catabolic pathways as an adaptive response to sugar starvation in *Arabidopsis thaliana*. Plant & Cell Physiology 59, 1363–1376. 29390157 10.1093/pcp/pcy005

[CIT0039] Hochberg Y, Benjamini Y 1990 More powerful procedures for multiple significance testing. Statistics in Medicine 9, 811–818. 2218183 10.1002/sim.4780090710

[CIT0040] Howard TP, Metodiev M, Lloyd JC, Raines CA 2008 Thioredoxin-mediated reversible dissociation of a stromal multiprotein complex in response to changes in light availability. Proceeding of the National Academy of Sciences, USA 105, 4056–4061. 10.1073/pnas.0710518105 PMC2268787 18322016

[CIT0041] Hudson AO, Bless C, Macedo P, Chatterjee SP, Singh BK, Gilvarg C, Leustek T 2005 Biosynthesis of lysine in plants: evidence for a variant of the known bacterial pathways. Biochimica et Biophysica Acta 172, 127–136. 10.1016/j.bbagen.2004.09.008 15652176

[CIT0042] Hudson AO, Singh BK, Leustek T, Gilvarg C 2006 An *LL*-diaminopimelate aminotransferase defines a novel variant of the lysine biosynthesis pathway in plants. Plant Physiology 140, 292–301. 16361515 10.1104/pp.105.072629 PMC1326051

[CIT0043] Irizarry RA, Hobbs B, Collin F, Beazer-Barclay YD, Antonellis KJ, Scherf U, Speed TP 2003 Exploration, normalization, and summaries of high density oligonucleotide array probe level data. Biostatistics 4, 249–264. 12925520 10.1093/biostatistics/4.2.249

[CIT0044] Ishizaki K, Schauer N, Larson TR, Graham IA, Fernie AR, Leaver CJ 2006 The mitochondrial electron transfer flavoprotein complex is essential for survival of *Arabidopsis* in extended darkness. The Plant Journal 47, 751–760. 16923016 10.1111/j.1365-313X.2006.02826.x

[CIT0045] Izumi M, Hidema J, Makino A, Ishida H 2013 Autophagy contributes to nighttime energy availability for growth in Arabidopsis. Plant Physiology 161, 1682–1693. 23457226 10.1104/pp.113.215632 PMC3613448

[CIT0046] Jones-Held S, Ambrozevicius LP, Campbell M, Drumheller B, Harrington E, Leustek T 2012 Two *Arabidopsis thaliana* dihydrodipicolinate synthases, DHDPS1 and DHDPS2, are unequally redundant. Functional Plant Biology 39, 1058–1067. 10.1071/FP12169 32480855

[CIT0047] Karchi H, Shaul O, Galili G 1994 Lysine synthesis and catabolism are coordinately regulated during tobacco seed development. Proceeding of the National Academy of Sciences, USA 91, 2577–2581. 10.1073/pnas.91.7.2577 PMC43412 8146157

[CIT0048] Kirma M, Araújo WL, Fernie AR, Galili G 2012 The multifaceted role of aspartate-family amino acids in plant metabolism. Journal of Experimental Botany 63, 4995–5001. 22516796 10.1093/jxb/ers119

[CIT0049] Knill T, Schuster J, Reichelt M, Gershenzon J, Binder S 2008 Arabidopsis branched-chain aminotransferase 3 functions in both amino acid and glucosinolate biosynthesis. Plant Physiology 146, 1028–1039. 18162591 10.1104/pp.107.111609 PMC2259058

[CIT0050] Kopka J, Schauer N, Krueger S, et al 2005 GMD@CSB.DB: the Golm metabolome database. Bioinformatics 21, 1635–1638. 15613389 10.1093/bioinformatics/bti236

[CIT0051] Lehmann M, Schwarzländer M, Obata T, et al 2009 The metabolic response of *Arabidopsis* roots to oxidative stress is distinct from that of heterotrophic cells in culture and highlights a complex relationship between the levels of transcripts, metabolites, and flux. Molecular Plant 2, 390–406. 19825624 10.1093/mp/ssn080

[CIT0052] Lehmeier CA, Lattanzi FA, Schäufele R, Wild M, Schnyder H 2008 Root and shoot respiration of perennial ryegrass are supplied by the same substrate pools: assessment by dynamic ^13^C labeling and compartmental analysis of tracer kinetics. Plant Physiology 148, 1148–1158. 18715953 10.1104/pp.108.127324 PMC2556832

[CIT0053] Less H, Angelovici R, Tzin V, Galili G 2011 Coordinated gene networks regulating *Arabidopsis* plant metabolism in response to various stresses and nutritional cues. The Plant Cell 23, 1264–1271. 21487096 10.1105/tpc.110.082867 PMC3101534

[CIT0054] Less H, Galili G 2008 Principal transcriptional programs regulating plant amino acid metabolism in response to abiotic stresses. Plant Physiology 147, 316–330. 18375600 10.1104/pp.108.115733 PMC2330312

[CIT0055] Less H, Galili G 2009 Coordinations between gene modules control the operation of plant amino acid metabolic networks. BMC Systems Biology 3, 14. 19171064 10.1186/1752-0509-3-14 PMC2646696

[CIT0056] Lisec J, Schauer N, Kopka J, Willmitzer L, Fernie AR 2006 Gas chromatography mass spectrometry-based metabolite profiling in plants. Nature Protocols 1, 387–396. 17406261 10.1038/nprot.2006.59

[CIT0057] Livak KJ, Schmittgen TD 2001 Analysis of relative gene expression data using real-time quantitative PCR and the 2(−ΔΔ*C*(T)) method. Methods 25, 402–408. 10.1006/meth.2001.1262 11846609

[CIT0058] Luedemann A, Strassburg K, Erban A, Kopka J 2008 TagFinder for the quantitative analysis of gas chromatography–mass spectrometry (GC-MS)-based metabolite profiling experiments. Bioinformatics 24, 732–737. 18204057 10.1093/bioinformatics/btn023

[CIT0059] Majumdar R, Shao L, Minocha R, Long S, Minocha SC 2013 Ornithine: the overlooked molecule in the regulation of polyamine metabolism. Plant & Cell Physiology 54, 990–1004. 23574701 10.1093/pcp/pct053

[CIT0060] Marri L, Trost P, Pupillo P, Sparla F 2005 Reconstitution and properties of the recombinant glyceraldehyde-3-phosphate dehydrogenase/CP12/phosphoribulokinase supramolecular complex of Arabidopsis. Plant Physiology 139, 1433–1443. 16258009 10.1104/pp.105.068445 PMC1283778

[CIT0061] Marri L, Zaffagnini M, Collin V, Issakidis-Bourguet E, Lemaire SD, Pupillo P, Sparla F, Miginiac-Maslow M, Trost P 2009 Prompt and easy activation by specific thioredoxins of calvin cycle enzymes of *Arabidopsis thaliana* associated in the GAPDH/CP12/PRK supramolecular complex. Molecular Plant 2, 259–269. 19825612 10.1093/mp/ssn061

[CIT0062] McCoy AJ, Adams NE, Hudson AO, Gilvarg C, Leustek T, Maurelli AT 2006 *L*,*L*-diaminopimelate aminotransferase, a trans-kingdom enzyme shared by *Chlamydia* and plants for synthesis of diaminopimelate/lysine. Proceedings of the National Academy of Sciences, USA 103, 17909–17914. 10.1073/pnas.0608643103 PMC1693846 17093042

[CIT0063] Michaeli S, Fromm H 2015 Closing the loop on the GABA shunt in plants: are GABA metabolism and signaling entwined?Frontiers in Plant Science 6, 419. 26106401 10.3389/fpls.2015.00419 PMC4460296

[CIT0064] Millar AH, Whelan J, Soole KL, Day DA 2011 Organization and regulation of mitochondrial respiration in plants. Annual Review of Plant Biology 62, 79–104. 10.1146/annurev-arplant-042110-103857 21332361

[CIT0065] Moller IM, Kristensen BK 2004 Protein oxidation in plant mitochondria as a stress indicator. Photochemical & Photobiological Sciences 3, 730–735. 15295627 10.1039/b315561g

[CIT0066] Nunes-Nesi A, Carrari F, Gibon Y, Sulpice R, Lytovchenko A, Fisahn J, Graham J, Ratcliffe RG, Sweetlove LJ, Fernie AR 2007 Deficiency of mitochondrial fumarase activity in tomato plants impairs photosynthesis via an effect on stomatal function. The Plant Journal 50, 1093–1106. 17461782 10.1111/j.1365-313X.2007.03115.x

[CIT0067] Peng C, Uygun S, Shiu SH, Last RL 2015 The impact of the branched-chain ketoacid dehydrogenase complex on amino acid homeostasis in Arabidopsis. Plant Physiology 169, 1807–1820. 25986129 10.1104/pp.15.00461 PMC4634046

[CIT0068] Piques M, Schulze WX, Höhne M, Usadel B, Gibon Y, Rohwer J, Stitt M 2009 Ribosome and transcript copy numbers, polysome occupancy and enzyme dynamics in *Arabidopsis*. Molecular Systems Biology 5, 314. 19888209 10.1038/msb.2009.68 PMC2779082

[CIT0069] Plaxton WC, Podesta FE 2006 The functional organization and control of plant respiration. Critical Review in Plant Science 25, 159–198.

[CIT0070] Ragel P, Streb S, Feil R, Sahrawy M, Annunziata MG, Lunn JE, Zeeman S, Mérida Á 2013 Loss of starch granule initiation has a deleterious effect on the growth of Arabidopsis plants due to an accumulation of ADP-glucose. Plant Physiology 163, 75–85. 23872660 10.1104/pp.113.223420 PMC3762666

[CIT0071] Rasmusson AG, Fernie AR, van Dongen JT 2009 Alternative oxidase: a defence against metabolic fluctuations?Physiologia Plantarum 137, 371–382. 19558416 10.1111/j.1399-3054.2009.01252.x

[CIT0072] Rate DN, Greenberg JT 2001 The *Arabidopsis* aberrant growth and death2 mutant shows resistance to *Pseudomonas syringae* and reveals a role for NPR1 in suppressing hypersensitive cell death. The Plant Journal 27, 203–211. 11532166 10.1046/j.0960-7412.2001.1075umedoc.x

[CIT0073] Raven JA 2012 Protein turnover and plant RNA and phosphorus requirements in relation to nitrogen fixation. Plant Science 188–189, 25–35. 10.1016/j.plantsci.2012.02.010 22525241

[CIT0074] Riewe D, Koohi M, Lisec J, Pfeiffer M, Lippmann R, Schmeichel J, Willmitzer L, Altmann T 2012 A tyrosine aminotransferase involved in tocopherol synthesis in Arabidopsis. The Plant Journal 71, 850–859. 22540282 10.1111/j.1365-313X.2012.05035.x

[CIT0075] Ruuska SA, Ohlrogge JB 2001 Protocol for small-scale RNA isolation and transcriptional profiling of developing *Arabidopsis* seeds. Biotechniques 31, 752, 754, 756–758. 11680703 10.2144/01314bm08

[CIT0076] Sarrobert C, Thibaud MC, Contard-David P, Gineste S, Bechtold N, Robaglia C, Nussaume L 2000 Identification of an *Arabidopsis thaliana* mutant accumulating threonine resulting from mutation in a new dihydrodipicolinate synthase gene. The Plant Journal 24, 357–367. 11069709 10.1046/j.1365-313x.2000.00884.x

[CIT0077] Schauer N, Steinhauser D, Strelkov S, et al 2005 GC-MS libraries for the rapid identification of metabolites in complex biological samples. FEBS Letters 579, 1332–1337. 15733837 10.1016/j.febslet.2005.01.029

[CIT0078] Schertl P, Cabassa C, Saadallah K, Bordenave M, Savouré A, Braun HP 2014 Biochemical characterization of proline dehydrogenase in *Arabidopsis* mitochondria. The FEBS Journal 281, 2794–2804. 24751239 10.1111/febs.12821

[CIT0079] Serrato AJ, Fernández-Trijueque J, Barajas-López JD, Chueca A, Sahrawy M 2013 Plastid thioredoxins: a “one-for-all” redox-signaling system in plants. Frontiers in Plant Science 4, 463. 24319449 10.3389/fpls.2013.00463 PMC3836485

[CIT0080] Shevchenko A, Tomas H, Havlis J, Olsen JV, Mann M 2006 In-gel digestion for mass spectrometric characterization of proteins and proteomes. Nature Protocols 1, 2856–2860. 17406544 10.1038/nprot.2006.468

[CIT0081] Sienkiewicz-Porzucek A, Sulpice R, Osorio S, Krahnert I, Leisse A, Urbanczyk-Wochniak E, Hodges M, Fernie AR, Nunes-Nesi A 2010 Mild reductions in mitochondrial NAD-dependent isocitrate dehydrogenase activity result in altered nitrate assimilation and pigmentation but do not impact growth. Molecular Plant 3, 156–173. 20035036 10.1093/mp/ssp101 PMC2807928

[CIT0082] Song JT, Lu H, Greenberg JT 2004 Divergent roles in *Arabidopsis thaliana* development and defense of two homologous genes, and *AGD2-LIKE DEFENSE RESPONSE PROTEIN1*, encoding novel aminotransferases. The Plant Cell 16, 353–366. 14729919 10.1105/tpc.019372 PMC341909

[CIT0083] Stitt M, Müller C, Matt P, Gibon Y, Carillo P, Morcuende R, Scheible W-R, Krapp A 2002 Steps towards an integrated view of nitrogen metabolism. Journal of Experimental Botany 53, 959–970. 11912238 10.1093/jexbot/53.370.959

[CIT0084] Stitt M, Zeeman SC 2012 Starch turnover: pathways, regulation and role in growth. Current Opinion in Plant Biology 15, 282–292. 22541711 10.1016/j.pbi.2012.03.016

[CIT0085] Struys EA, Jakobs C 2010 Metabolism of lysine in α-aminoadipic semialdehyde dehydrogenase-deficient fibroblasts: evidence for an alternative pathway of pipecolic acid formation. FEBS Letters 584, 181–186. 19932104 10.1016/j.febslet.2009.11.055

[CIT0086] Sulpice R, Pyl E-T, Ishihara H, et al 2009 Starch as a major integrator in the regulation of plant growth. Proceeding of the National Academy of Science, USA 106, 10348–10353. 10.1073/pnas.0903478106 PMC2693182 19506259

[CIT0087] Sweetlove LJ, Fernie AR 2005 Regulation of metabolic networks: understanding metabolic complexity in the systems biology era. New Phytologist 168, 9–24. 16159317 10.1111/j.1469-8137.2005.01513.x

[CIT0088] Ufaz S, Galili G 2008 Improving the content of essential amino acids in crop plants: goals and opportunities. Plant Physiology 147, 954–961. 18612072 10.1104/pp.108.118091 PMC2442549

[CIT0089] Usadel B, Nagel A, Steinhauser D, et al 2006 PageMan: an interactive ontology tool to generate, display, and annotate overview graphs for profiling experiments. BMC Bioinformatics 7, 535. 17176458 10.1186/1471-2105-7-535 PMC1766370

[CIT0090] Wang S, Blumwald E 2014 Stress-induced chloroplast degradation in *Arabidopsis* is regulated via a process independent of autophagy and senescence-associated vacuoles. The Plant Cell 26, 4875–4888. 25538186 10.1105/tpc.114.133116 PMC4311210

[CIT0091] Wang W, Galili G 2016 Transgenic high-lysine rice—a realistic solution to malnutrition?Journal of Experimental Botany 67, 4009–4011. 27402615 10.1093/jxb/erw254 PMC5301943

[CIT0092] Weigelt K, Küster H, Radchuk R, Müller M, Weichert H, Fait A, Fernie AR, Saalbach I, Weber H 2008 Increasing amino acid supply in pea embryos reveals specific interactions of N and C metabolism, and highlights the importance of mitochondrial metabolism. The Plant Journal 55, 909–926. 18494854 10.1111/j.1365-313X.2008.03560.x

[CIT0093] Wulff-Zottele C, Gatzke N, Kopka J, Orellana A, Hoefgen R, Fisahn J, Hesse H 2010 Photosynthesis and metabolism interact during acclimation of *Arabidopsis thaliana* to high irradiance and sulphur depletion. Plant, Cell & Environment 33, 1974–1988. 10.1111/j.1365-3040.2010.02199.x 20573050

[CIT0094] Zhu X, Galili G 2003 Increased lysine synthesis coupled with a knockout of its catabolism synergistically boosts lysine content and also transregulates the metabolism of other amino acids in Arabidopsis seeds. The Plant Cell 15, 845–853. 12671081 10.1105/tpc.009647 PMC152333

