# Supplementary material for: An L,L-diaminopimelate aminotransferase mutation leads to metabolic shifts and growth inhibition in Arabidopsis
Source: J Exp Bot. 2018 Sep 12;69(22):5489–506. doi: 10.1093/jxb/ery325 (PMC6255705; doi:10.1093/jxb/ery325)
Supplement: Supplementary Figures S1-S5 [file ery325_suppl_supplementary_figures_s1-s5.pdf]

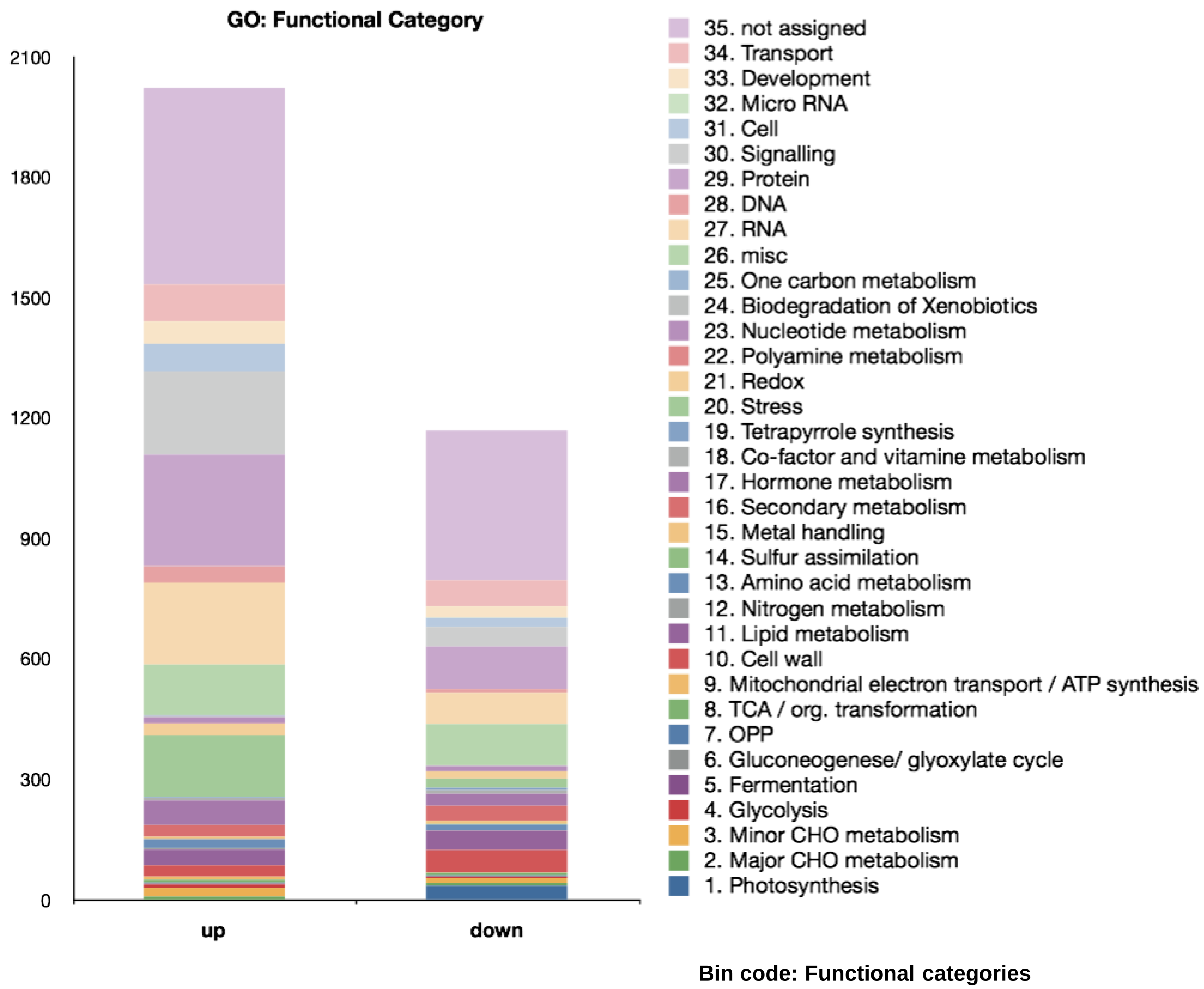

Supplementary Fig. S1. Bar chart of functional categories of differentially changed genes in *dapat* mutants (PageMan analysis; Usadel et al., 2006).

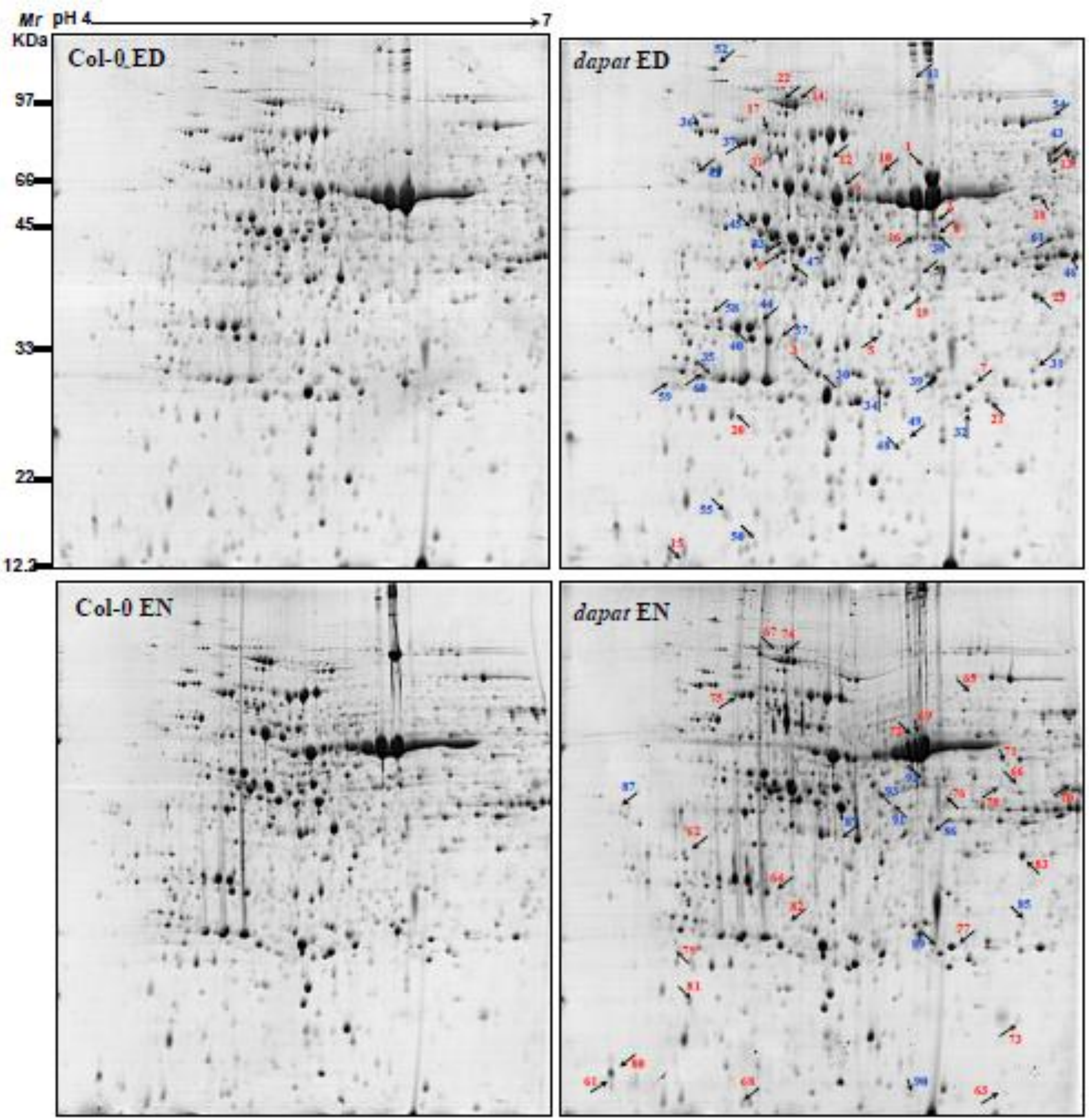

Supplementary Fig. S2. 2D gel maps of rosette of Arabidopsis in two points: (A) end of day and (B) end of night (ED). Proteins were extracted by phenol follow methanol/ammonium acetate precipitation and separated by IEF/SDS-PAGE then stained with CBB-250G (see material and methods). An equal amount (850µg) of total protein was load on each gel strip and resolved of 18 cm length in 4-7 pH linear gradient range.

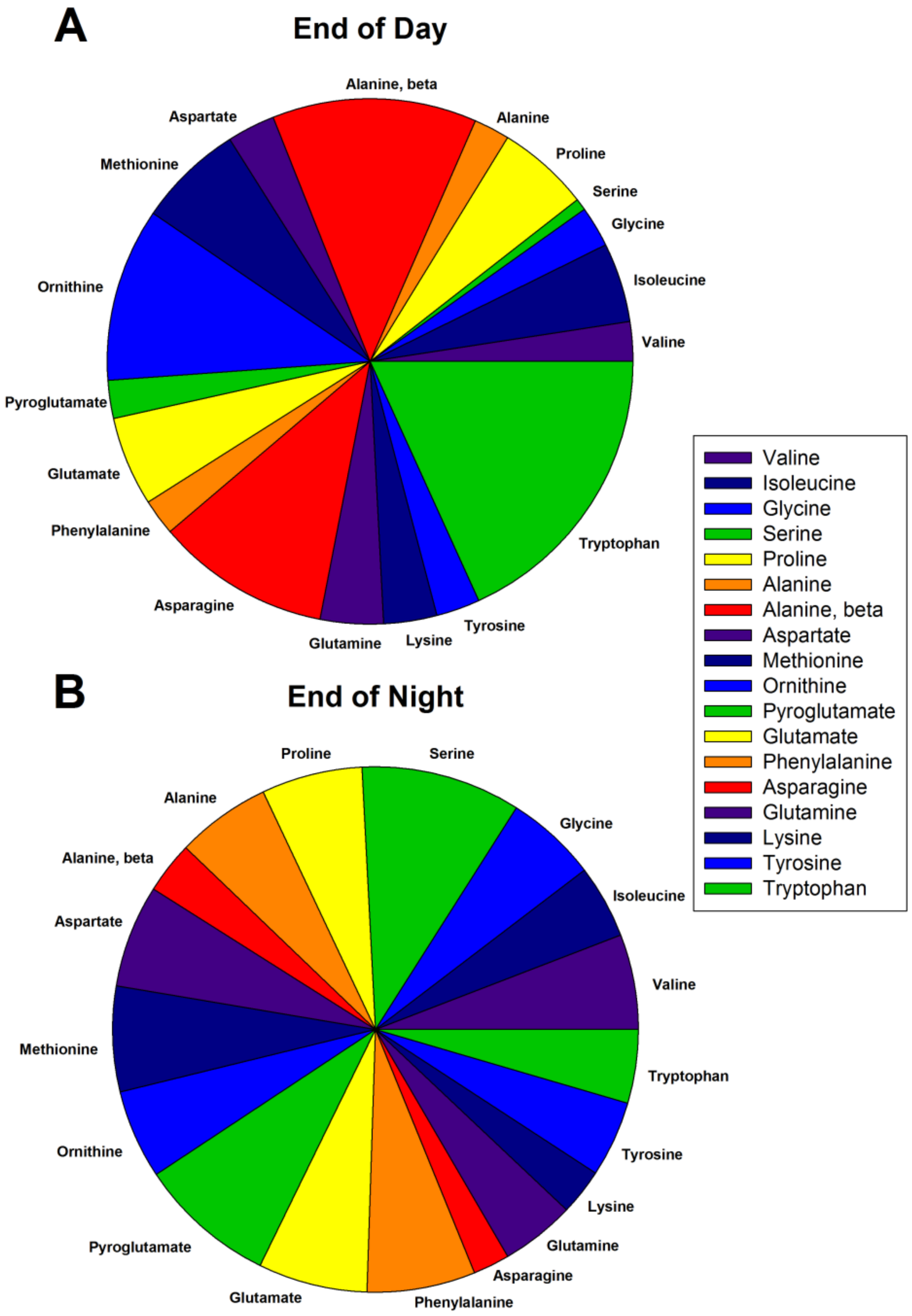

Supplementary Fig. S3. Pie chart comparing the impact of each amino acid on amino acid pools between *dapat* and wild-type along diurnal cycle: (A) at the end of day (ED) and (B) at the end of night (EN).

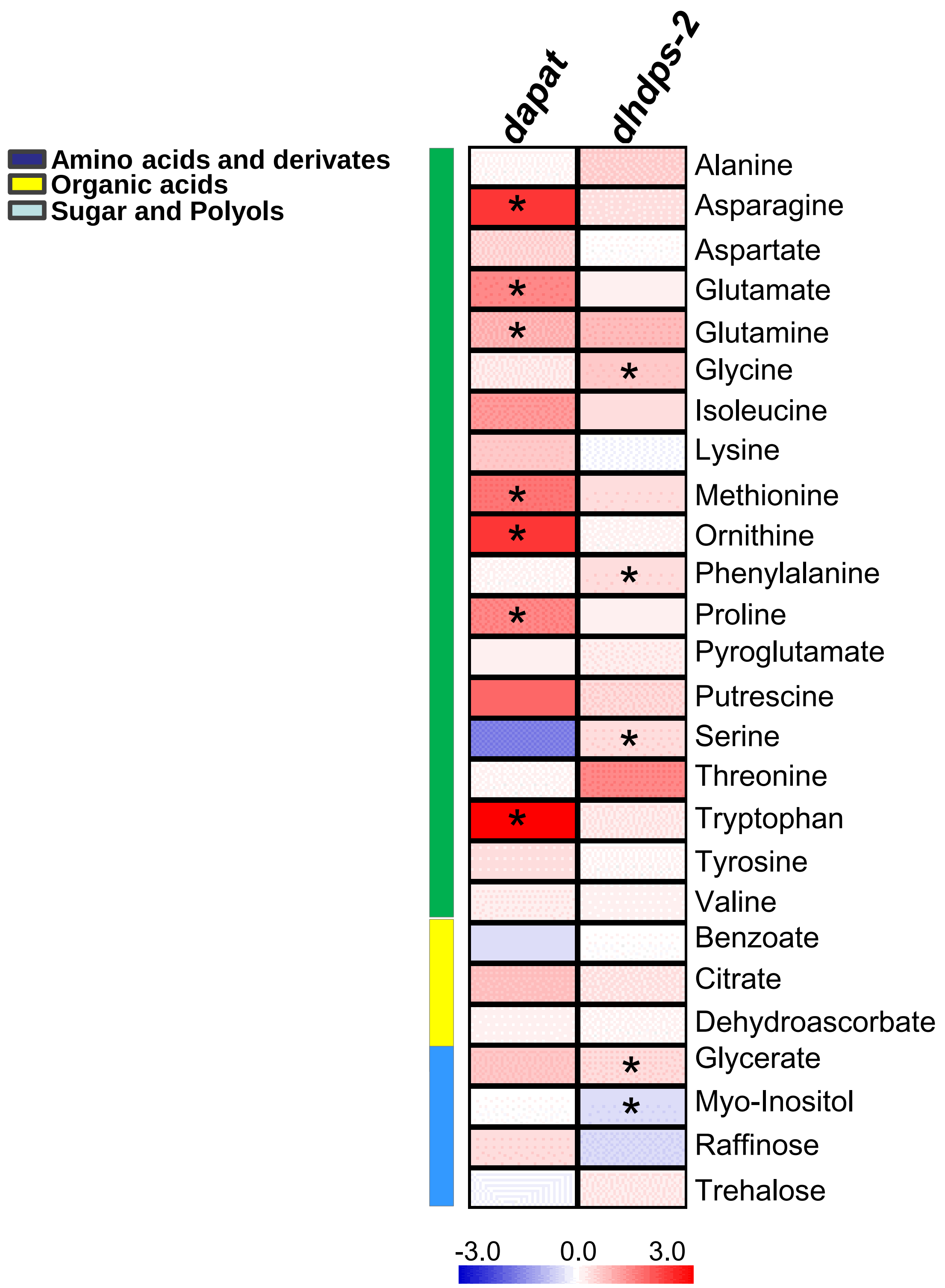

Supplementary Fig. S4. Heatmap comparison of GCMS metabolite profiling between *dapat* and *dhdps2*. Data are normalized with respect to the mean response calculated for WT, Col-0 and WS to dapat and dhdps2, respectively.. The colour code of the heat map is given at the log scale close to the diagrams. Values are average of 4 independent biological replicates. Color squares designate that significantly differ (*P* <0.05) by the t-test.

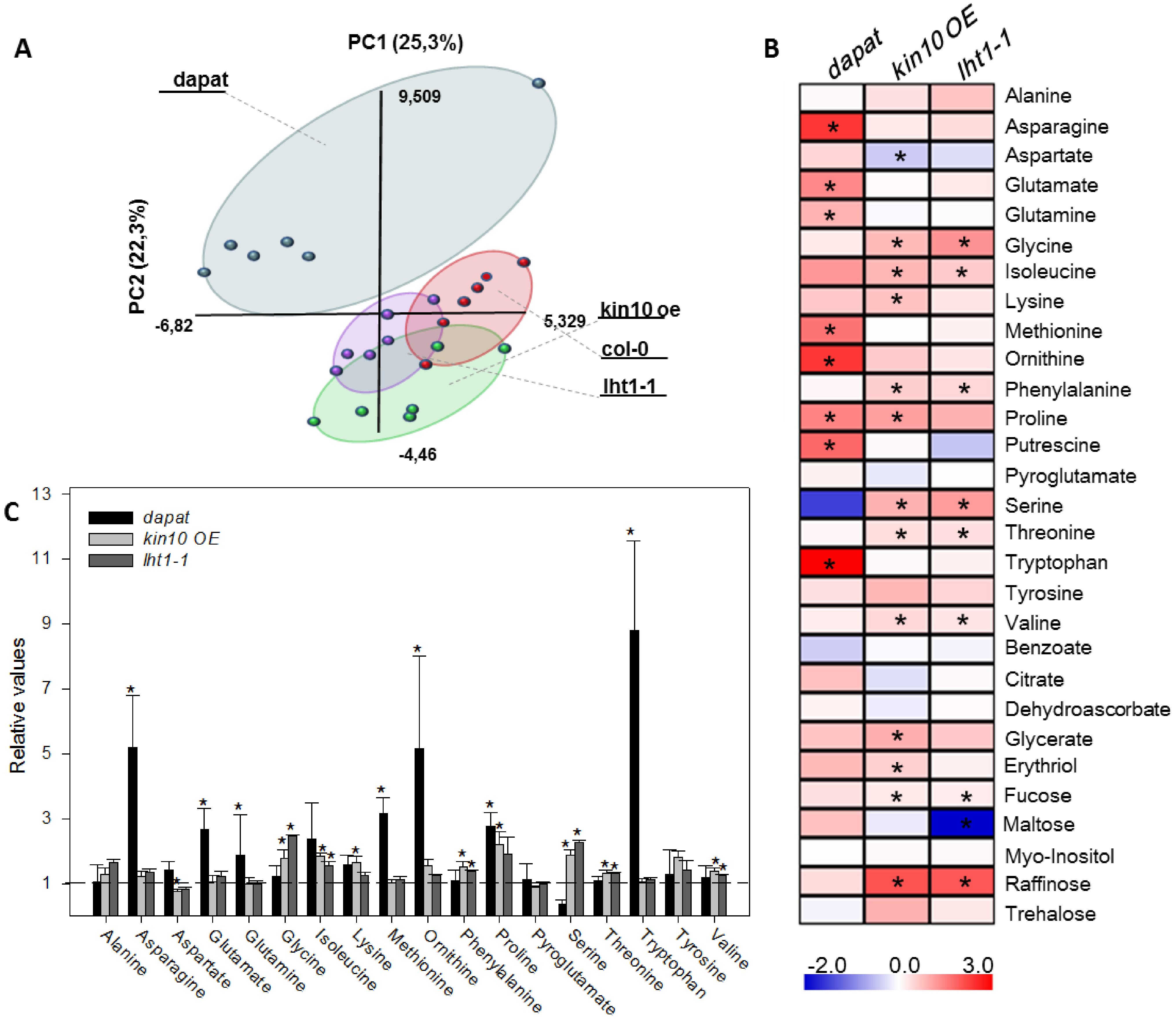

Supplementary Fig. S5. Differences among *dapat, kin10* OE and *lht1-1* mutants. (A) Principal Component Analysis was performed using the metabolic data of *dapa*t, *kin10 OE* and *lht1-1* mutants. The respective mutants symbols are indicated in the figure. (B) Log2 values of the relative metabolic content are presented as a heat map. Significant differences compared with the wild type following Student's t test are denoted by one asterisk ($P < 0.05$) (C) Relative levels of amino acids compared with the wild type. Data are 5 independent biological replicates presented with SE; significant differences following Student's t test are denoted by an asterisk ($P < 0.05$)
